# Novel Potent and Selective Agonists of the GPR55 Receptor Based on the 3-Benzylquinolin-2(1*H*)-One Scaffold

**DOI:** 10.3390/ph15070768

**Published:** 2022-06-21

**Authors:** Costanza Ceni, Michael J. Benko, Kawthar A. Mohamed, Giulio Poli, Miriana Di Stefano, Tiziano Tuccinardi, Maria Digiacomo, Massimo Valoti, Robert B. Laprairie, Marco Macchia, Simone Bertini

**Affiliations:** 1 Department of Pharmacy, University of Pisa, Via Bonanno Pisano 6, 56126 Pisa, Italy; costanza.ceni@phd.unipi.it (C.C.); giulio.poli@unipi.it (G.P.); miriana.distefano@phd.unipi.it (M.D.S.); tiziano.tuccinardi@unipi.it (T.T.); maria.digiacomo@unipi.it (M.D.); marco.macchia@unipi.it (M.M.); 2 Doctoral School in Life Sciences, University of Siena, Via Aldo Moro 2, 53100 Siena, Italy; 3 College of Pharmacy and Nutrition, University of Saskatchewan, Saskatoon, SK S7N 5E5, Canada; michael.benko@usask.ca (M.J.B.); kam913@mail.usask.ca (K.A.M.); 4 Department of Life Sciences, University of Siena, Via Aldo Moro 2, 53100 Siena, Italy; massimo.valoti@unisi.it

**Keywords:** GPR55, GPR55 agonist, GPR55 modulator, p-ERK activation assay, quinolin-2-one, benzylquinolin-2-one

## Abstract

A growing body of evidence underlines the crucial role of GPR55 in physiological and pathological conditions. In fact, GPR55 has recently emerged as a therapeutic target for several diseases, including cancer and neurodegenerative and metabolic disorders. Several lines of evidence highlight GPR55′s involvement in the regulation of microglia-mediated neuroinflammation, although the exact molecular mechanism has not been yet elucidated. Nevertheless, there are only a limited number of selective GPR55 ligands reported in the literature. In this work, we designed and synthesized a series of novel GPR55 ligands based on the 3-benzylquinolin-2(1*H*)-one scaffold, some of which showed excellent binding properties (with *K*_i_ values in the low nanomolar range) and almost complete selectivity over cannabinoid receptors. The full agonist profile of all the new derivatives was assessed using the p-ERK activation assay and a computational study was conducted to predict the key interactions with the binding site of the receptor. Our data outline a preliminary structure–activity relationship (SAR) for this class of molecules at GPR55. Some of our compounds are among the most potent GPR55 agonists developed to date and could be useful as tools to validate this receptor as a therapeutic target.

## 1. Introduction

**Figure 1:**
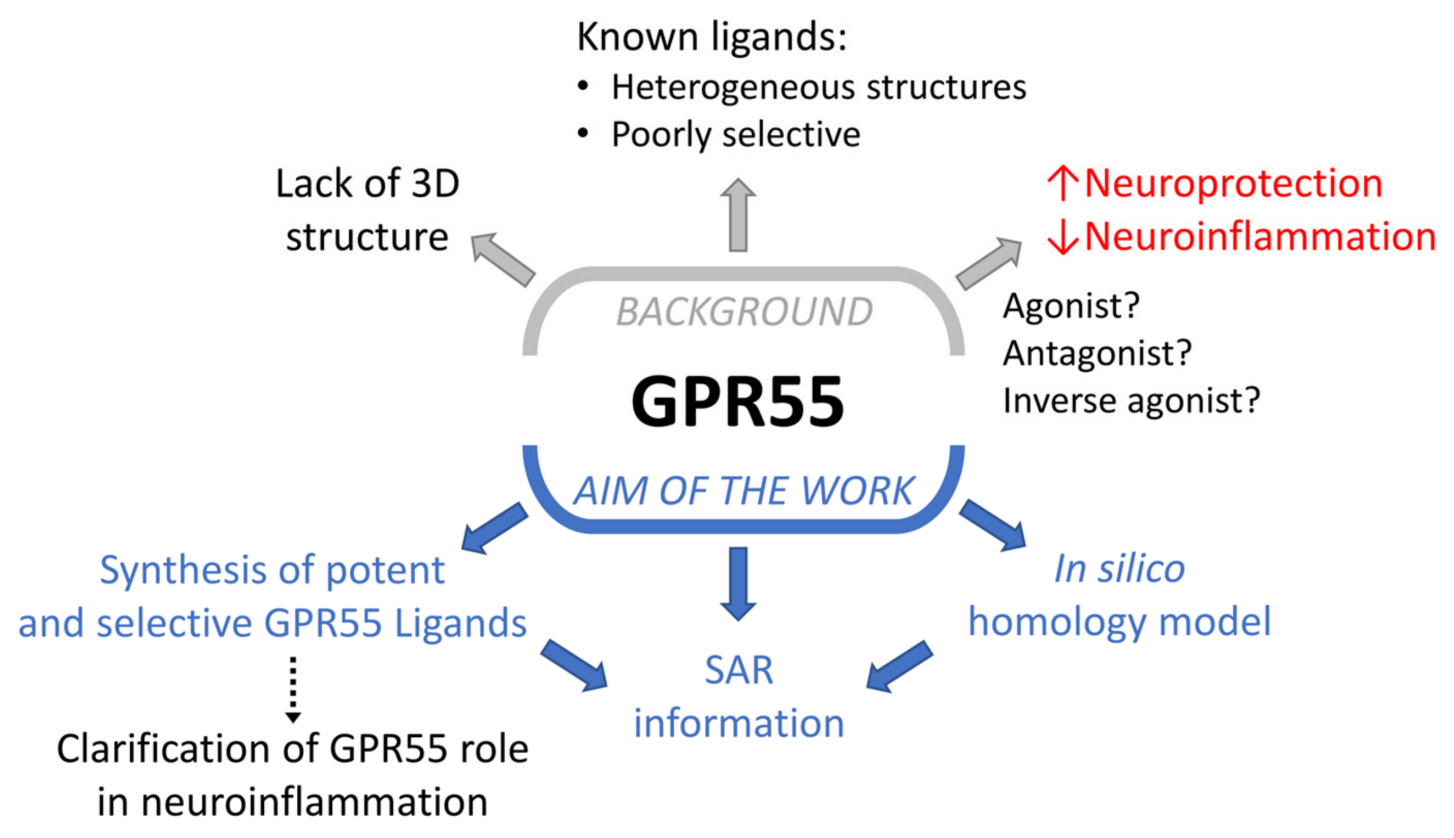
Background and aim of this work.

GPR55 is a class A G protein-coupled receptor (GPCR) whose endogenous agonist has been identified as L-α-lysophosphatidylinositol (LPI, Figure 1) [1,2]. It is a peculiar GPCR, since it does not bind classical G proteins (G_i_, G_s_, G_o_), but only G_q_ and G_12_, _13_, leading to activation of phospholipase C (PLC), directly or indirectly (via ROCK and RhoA signaling pathways, respectively) [2]. This results in the induction of inositol 1,4,5-trisphosphate (IP_3_), with the consequent release of intracellular calcium [3,4,5,6]. Furthermore, several studies have confirmed its involvement in the activation of ERK1/2 and the MAP-kinase cascade [7,8,9], as well as in β-arrestin translocation [1,8].

GPR55 was cloned more than twenty years ago [10] and initially deorphanized as a cannabinoid receptor [11], but its categorization remains under debate. Despite having a low amino acid similarity with both CB1R (13.5%) and CB2R (14.4%), GPR55 has been found to interact with some cannabinoid ligands, including Δ^9^-tetrahydrocannabinol (Δ^9^-THC), CP55,940 and rimonabant (Figure 1) [12]. GPR55 is expressed in several peripheral organs, such as the gastrointestinal tract [13], pancreas [14], bone tissue [3], vascular endothelial cells [15] and immune system [16], representing a novel therapeutic target for the treatment of diabetes [14], osteoporosis [3] and cancer [17,18]. It is also highly expressed in microglia cells where it is involved in the regulation of neuroinflammatory processes, although its precise role has not yet been completely elucidated [19].

Recent studies reported a microglia-mediated neuroprotective effect following GPR55 activation both in an in vitro model of rat hippocampal slice cultures [20] and in a mouse model of Parkinson’s disease (PD) [21], corroborated by subsequent evidence that GPR55 agonists attenuate injury in neural stem cells [22]. Another recent study suggested an anti-neuroinflammatory effect mediated by GPR55 antagonists/inverse agonists in lipopolysaccharide (LPS)-activated primary microglial cells [23]. Given GPR55’s apparent role in neuroinflammation, excitotoxicity, oxidative stress and mitochondrial dysfunction closely linked to neurodegenerative processes [24,25], the development of novel GPR55 modulators could not only represent a key step to better understanding the precise role of this receptor in neuroprotection, but also provide a potential innovative therapeutic strategy for the treatment of neurodegenerative diseases with a neuroinflammatory background. Furthermore, given the limited number of selective GPR55 modulators reported to date, novel potent and selective GPR55 ligands could also represent potential tool compounds to provide important information about structural requirements for the interaction with GPR55’s binding site and to validate this receptor as a therapeutic target. Figure 1 schematically represents the background and the aim of this work.

**Figure 2:**
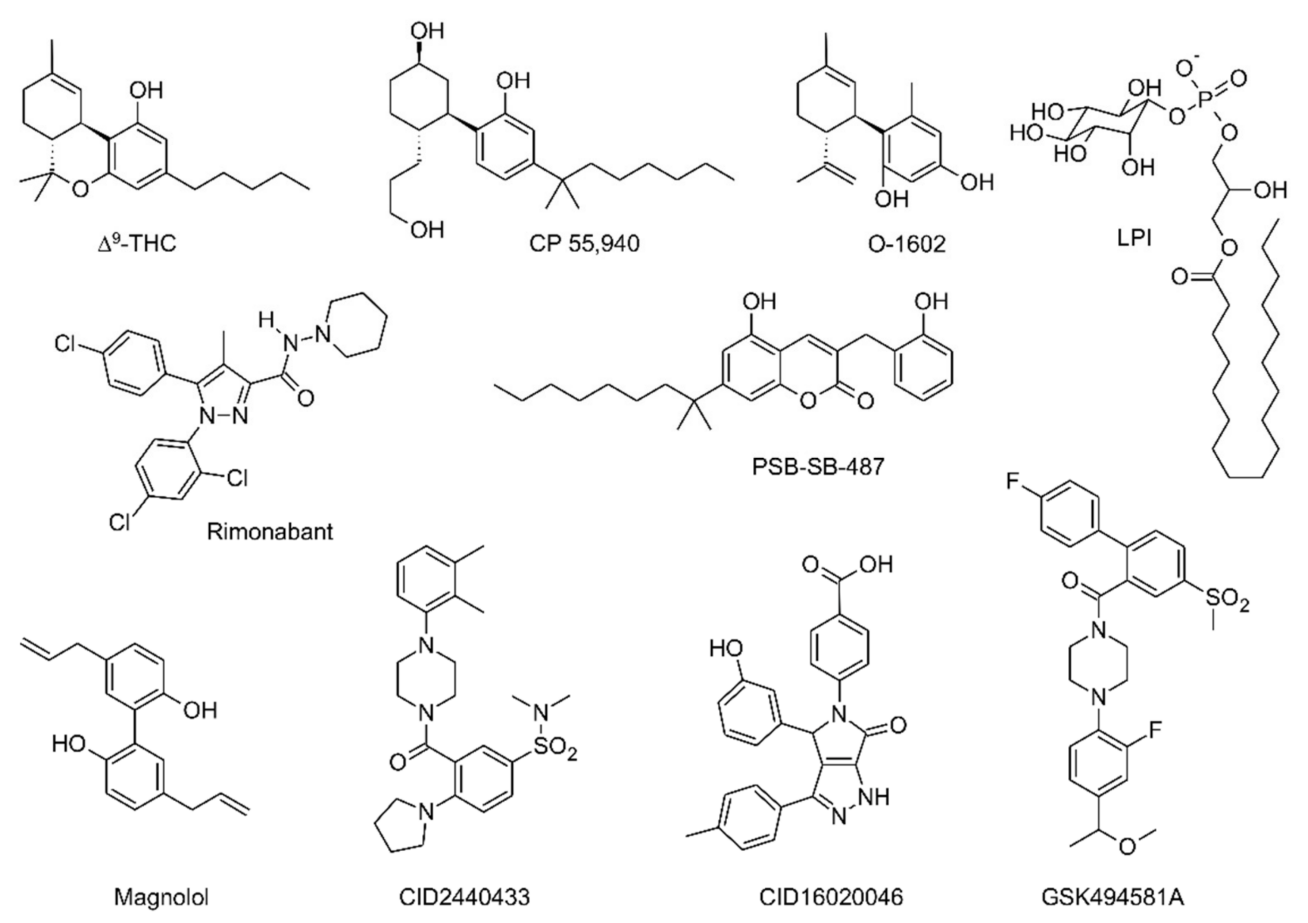
Chemical structures of representative GPR55 ligands.

GPR55 ligands known to date are structurally heterogeneous and, as mentioned above, in general poorly selective. In addition to phosphatidylinositols (e.g., LPI) and the aforementioned natural and synthetic cannabinoids (Δ^9^-THC, CP 55,940, rimonabant), other ligands have been identified, among which are O-1602 (a synthetic compound closely related to abnormal cannabidiol), some coumarin derivatives (e.g., PSB-SB-487), magnolol derivatives and other compounds bearing different scaffolds obtained from high-throughput screenings and other subsequent studies (e.g., CID2440433, CID16020046 and GSK494581A) [26]. The chemical structures of representative GPR55 ligands are shown in Figure 2.

**Figure 3:**
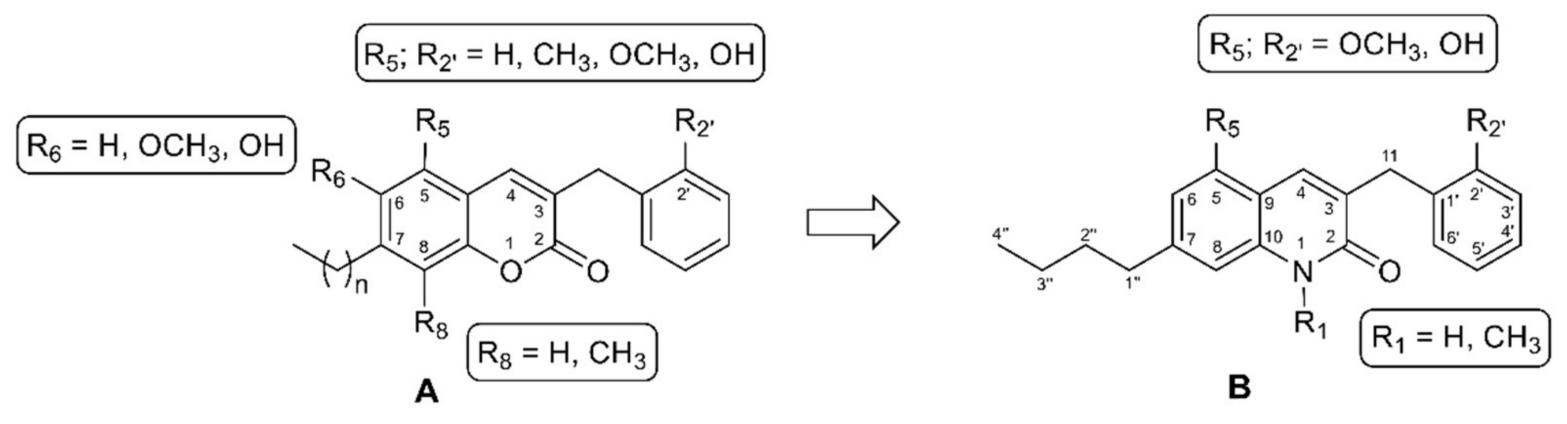
Structural modifications providing the novel series of 3-benzylquinolin-2(1*H*)-ones (**B**) from 3-benzylcoumarins (**A**).

Recently, a class of 3-benzylcoumarin derivatives (general structure **A**, Figure 3) has been reported, showing a GPR55 antagonist profile, evaluated through the β-arrestin assay, with IC_50_ values in the low micromolar range [12]. Starting from these derivatives, we decided to change the stereo-electronic properties of the central coumarin scaffold through the replacement of the lactone group with an amide moiety, with the aim of identifying novel potent and selective GPR55 modulators. In this work we thus report the synthesis and the biological evaluation of a series of variously substituted 3-benzylquinolin-2(1*H*)-ones **1**–**4**, with general structure **B**, (Figure 3, Table 1). Additional modifications concern *N*-methylation and the introduction of an *n*-butyl group at position 7 as a sidechain of intermediate length with respect to those reported for the coumarin derivatives (methyl, *iso*-propyl, heptyl, dimethyloctyl groups) [12]. Moreover, at positions 5 and 2′, we introduced hydroxyl or methoxy groups, taking into account that the insertion of small polar substituents at the same positions of the 3-benzylcoumarin scaffold led to the best results in terms of activity at GPR55 [12].

While the available data on GPR55 ligands have been essentially obtained through functional assays in transfected cells, the affinity of the compounds in this work has been evaluated via radioligand binding using [^3^H]CP55,940, which allowed for the estimation of *K*_i_ values. This assay builds on previous radioligand binding studies of GPR55 where CP55,940 displayed clear and specific affinity [11]. Some of the newly synthesized 3-benzylquinolin-2(1*H*)-ones showed excellent binding properties with *K*_i_ values in the low nanomolar range and almost complete selectivity over CB1R and CB2R. The p-ERK activation assay was used to evaluate the pharmacological profile of the synthesized compounds. Interestingly, all of them displayed a functional activity of full agonists of GPR55. Finally, a computational study was carried out to predict the key interactions of the ligands with the binding site of the GPR55 receptor.

## 2. Results

### 2.1. Chemistry

**Scheme 1:**
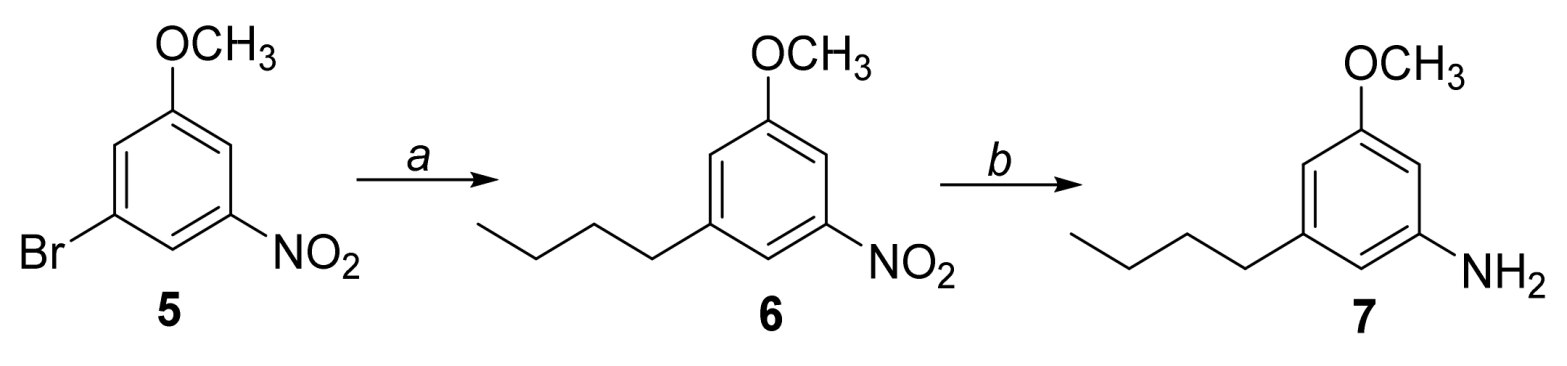
Reagents and conditions: (**a**) *n*-Butylboronic acid, Pd_2_(dba)_3_, Ruphos, *t-*BuONa, toluene/H_2_O (10:1), 80 °C, overnight; (**b**) Fe^0^, NH_4_Cl, EtOH/H_2_O (2:1), reflux, 3 h.

The amine intermediate **7** was prepared as reported in Scheme 1. The commercially available 3-bromo-5-nitroanisole **5** underwent a cross-coupling reaction with *n*-butylboronic acid in a mixture of toluene/H_2_O in the presence of Pd_2_(dba)_3_, Ruphos and *t*-BuONa, at 80 °C overnight, affording compound **6**. It was then dissolved in a mixture of EtOH/H_2_O and treated with iron powder and NH_4_Cl, at reflux for 3 h, to afford the intermediate **7**.

**Scheme 2:**
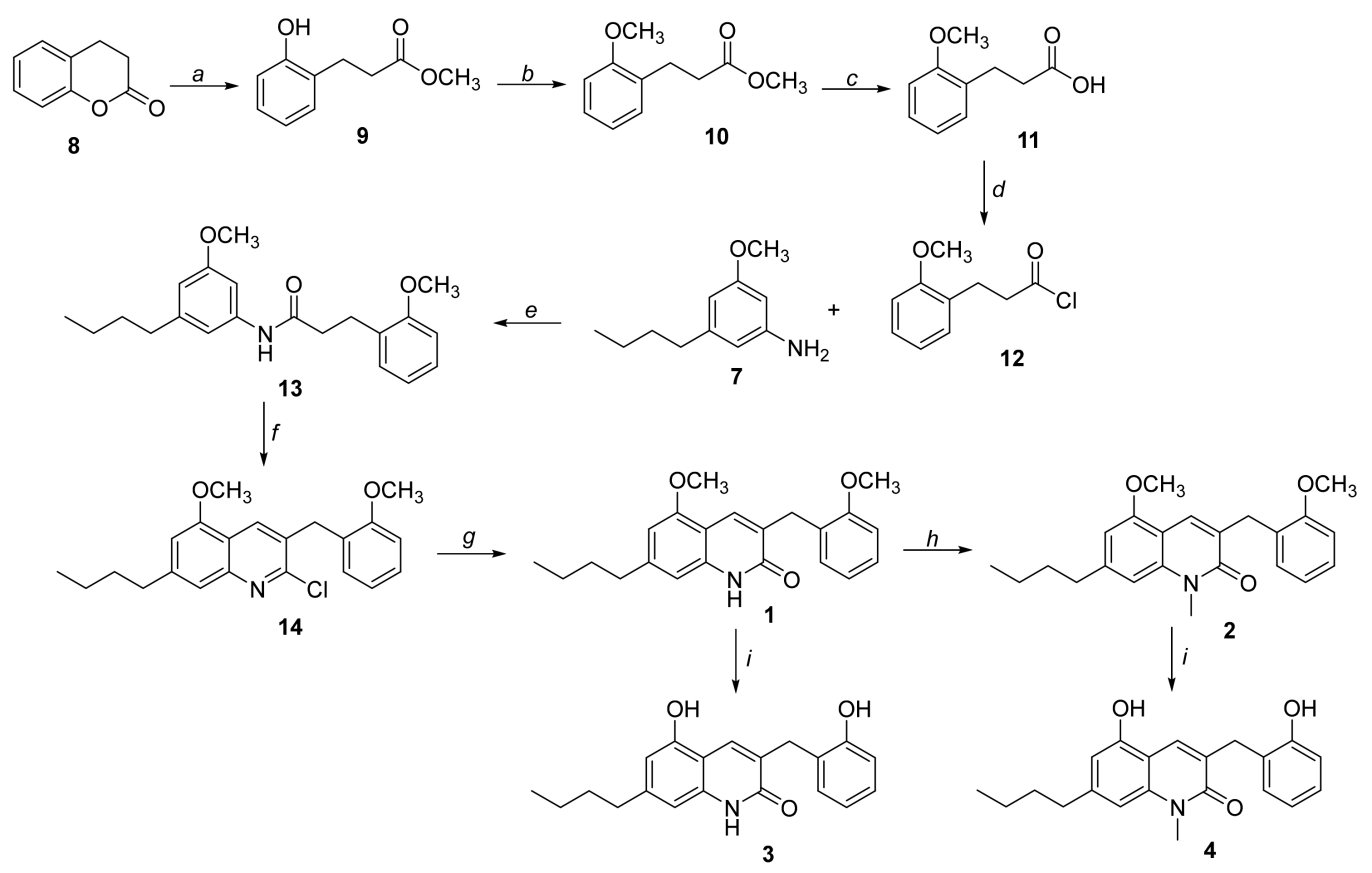
Reagents and conditions: (**a**) conc. H_2_SO_4_, dry MeOH, reflux, overnight; (**b**) CH_3_I, NaH, DMF, rt, overnight; (**c**) LiOH, EtOH/H_2_O (3:1), rt, 3 h; (**d**) SOCl_2_, toluene, reflux, 3 h; (**e**) Et_3_N, CH_2_Cl_2_, rt, overnight; (**f**) DMF, POCl_3_, 80 °C, overnight; (**g**) HCl 6 N, reflux, overnight; (**h**) NaH, CH_3_I, DMF, rt, overnight; (**i**) BBr_3_, DCM, rt, overnight.

The synthetic procedure leading to 3-benzylquinolin-2(1*H*)-ones **1**–**4** is reported in Scheme 2. The commercially available 3,4-dihydrocumarine **8** was treated with concentrated H_2_SO_4_ in anhydrous MeOH, affording compound **9**, which underwent a methylation reaction in the presence of CH_3_I and NaH (60% dispersion in mineral oil) in DMF, obtaining intermediate **10**. After the hydrolysis of the ester group with LiOH, compound **11** was treated with SOCl_2_ in toluene to afford intermediate **12**, which led to amide derivative **13** by reaction with compound **7** in the presence of Et_3_N in DCM. The cyclization of **13** with POCl_3_ and DMF (Vilsmeier-Haack type reaction) provided derivative **14,** which was separated from the other positional isomer formed in this reaction (5-*n*-butyl-2-chloro-7-methoxy-3-(2-methoxybenzyl)quinoline **15**, see Supplementary Materials, Figure S6) by flash chromatography (see the section “Materials and Methods” for separation conditions). The proper structures of these two isomers were assigned on the basis of the corresponding NOESY spectra (see Supplementary Materials, Figures S5 and S6) and of the chemical shift of the H_4_ signal (8.19 ppm for **14**; 7.92 ppm for **15**). The desired intermediate **14** was then treated with 6 N HCl, affording compound **1**. This was subjected to a methylation reaction with CH_3_I in DMF, in the presence of NaH (60% dispersion in mineral oil), affording derivative **2**. Subsequent reactions of compounds **1** or **2** with BBr_3_ (1 M in anhydrous DCM) afforded demethylated derivatives **3** and **4**, respectively.

### 2.2. [^3^H]CP55,940 Binding Assays

**Figure 4:**
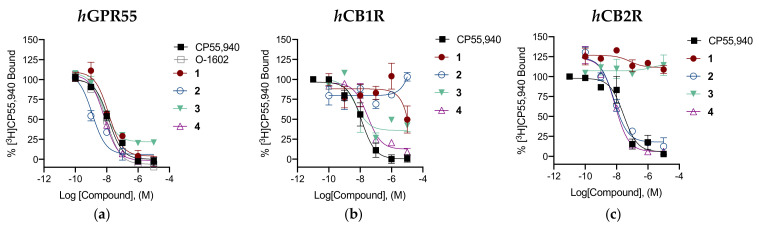
In vitro characterization of [^3^H]CP55,940 binding in the presence of compounds **1**–**4** at *h*GPR55 (**a**), *h*CB1R (**b**) or *h*CB2R (**c**). Data are expressed as % change from 1 nM [^3^H]CP55,940 binding and are means ± SEM (*n* = 3–4). All data were fit to a 3-parameter non-linear regression in GraphPad (v. 9.0). Analyses are presented in Table 1.

Compounds **1**–**4** were evaluated for GPR55 binding by incubating increasing concentrations of the compound with 1 nM [^3^H]CP55,940 in the presence of membrane preparations obtained from Chinese hamster ovary (CHO) cells expressing the human GPR55 receptor (25 µg protein). Two reference ligands were also tested in this assay: CP55,940, which is a full CBR agonist with high affinity toward GPR55; and O-1602, a synthetic compound closely related to abnormal cannabidiol (abn-CBD) that has been shown to activate the GPR55 receptor [11]. Furthermore, we investigated the selectivity of compounds **1**–**4** for GPR55 relative to human CB1R and CB2R using membranes from CHO cells expressing either of these receptors (25 µg protein) and 1 nM [^3^H]CP55,940. Radioligand binding affinity results are summarized in Table 1. GPR55, CB1R and CB2R competition binding graphs are shown in Figure 4.

### 2.3. Functional Assays

**Figure 5:**
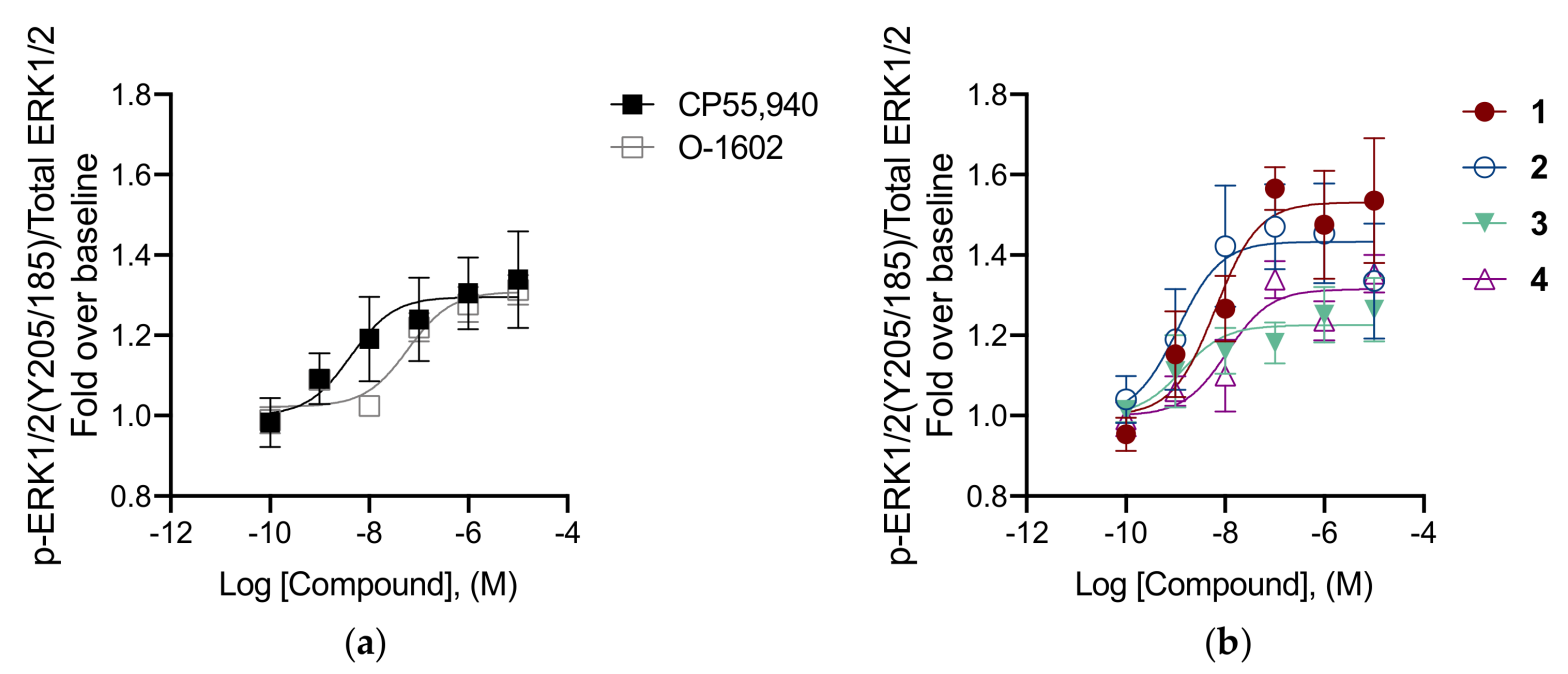
In vitro characterization of p-ERK1/2 in the presence of reference compounds CP55,940 and O-1602 (**a**) and of compounds **1**–**4** (**b**) at *h*GPR55. CHO cells expressing *h*GPR55 were treated with 0.1–10 µM compounds for 30 min. Data are expressed as fold over vehicle and are means ± SEM (*n* = 6 independent experiments performed in duplicate). All data were fit to a 3-parameter non-linear regression in GraphPad (v. 9.0). Analyses are presented in Table 2.

Downstream signaling by *h*GPR55 was investigated by quantifying p-ERK activation in the in-cell Western assay. Functional data for GPR55 are shown in Table 2. GPR55 p-ERK activation graphs are shown in Figure 5. 

### 2.4. Molecular Modeling

**Figure 6:**
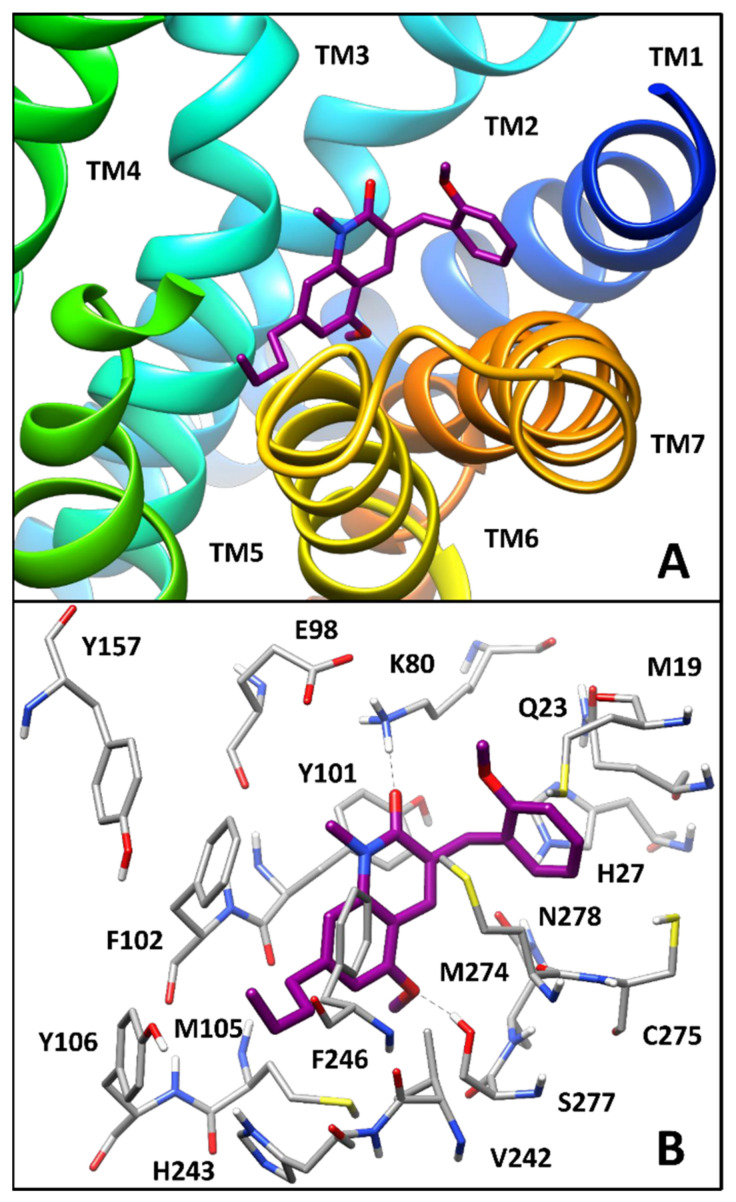
Minimized average structure of compound **2** within the binding site of the *h*GPR55 model. (**A**) The ligand (purple) and the different transmembrane domains of the receptor are shown; (**B**) the protein residues (gray) surrounding the ligand within the receptor binding site are shown, with ligand–protein H-bonds highlighted with black dashed lines.

In order to identify some structure-based hypotheses for explaining the preliminary SAR data emerging from the biological evaluation results obtained for the series of 3-benzyl-quinolin-2(1*H*)-ones, computational studies including homology modeling, molecular dynamics (MD) simulations, docking and binding free energy evaluations were employed for identifying the potential binding disposition of these compounds. Since no X-ray structure of human GPR55 (*h*GPR55) was currently available, a homology model of the receptor was initially built by using the X-ray structure of zebrafish lysophosphatidic acid (LPA) receptor LPA6 in complex with LPA (PDB code 5XSZ) [28] as a reference. In fact, among all proteins with available X-ray structure, this showed the highest sequence homology to *h*GPR55; moreover, the two receptors are activated by structurally analogous endogenous agonists. The *h*GPR55 homology model was embedded in a lipid bilayer composed of POPC molecules and the membrane–protein system was then refined through more than 230 ns of MD simulation in an explicit water environment (see the section “Materials and Methods” for details). The final MD-refined *h*GPR55 model was then used for molecular docking studies. Compound **2**, which showed the highest affinity for the receptor (*K*_i_ = 1.2 nM) and the highest potency (EC_50_ = 1.1 nM) was used as a representative ligand for the analysis. The compound was thus docked into the refined *h*GPR55 model using AUTODOCK 4.2 software [29] and employing a robust protocol; in particular, the side chains of Y3.32(101), M3.36(105), F6.48(239) and F6.55(246), which were found to be involved in agonist signaling at the GPR55 receptor [30], as well as F3.33(102) and M7.39(274), located at the entrance of the putative binding site, were treated as flexible during calculations. A representative (top-scored) binding mode was hence selected for each of the eight most populated clusters of solutions obtained using this protocol (see the section “Materials and Methods” for details); then, the corresponding ligand–protein complex was embedded in a phospholipid bilayer and analyzed through MD simulation using a 100 ns protocol analogous to that employed for *h*GPR55 model refinement (see Experimental for details). The stability of the studied complexes was initially analyzed based on the root-mean-square deviation (RMSD) of the ligand disposition during the MD, with respect to the initial binding pose predicted by docking. As reported in Table S1, three out of the eight complexes (namely **C2**, **C3** and **C8**) demonstrated a remarkable stability, since the average value of ligand RMSD during the simulation was far below 2.0 Å; on the contrary, complexes **C1**, **C5** and **C6** showed average ligand RMSD values between 4.0 and 5.3 Å, thus indicating that the binding mode of the compound was not well maintained during the MD. Finally, complexes **C4** and **C7** showed intermediate stability (average ligand RMSD of 2.1 and 2.2 Å, respectively). In order to assess the reliability of the various ligand binding poses from a quantitative point of view, all analyzed complexes were then subjected to ligand–protein binding free energy evaluations, performed based on the last 50 ns of MD simulation, using the Molecular Mechanics–Poisson–Boltzmann Surface Area (MM-PBSA) approach. Complex **C2**, which showed one of the lowest values of ligand RMSD during the whole MD simulation (average RMSD = 1.3 Å), was also suggested by the MM-PBSA analysis as the most energetically favorable one, since its predicted binding free energy was about 6 to 12 kcal/mol higher than that estimated for the other complexes (Table S2). Therefore, complex **C2** was identified as the most reliable one among the analyzed *h*GPR55-ligand complexes predicted for compound **2**, and the corresponding binding mode assumed by the ligand as the most reliable disposition of the inhibitor within the receptor. As shown in Figure 6, the ligand assumes an L-shaped binding conformation, in which the oxoquinoline core is essentially placed between two transmembrane domains, TM3 from one side and TM7 from the other, the butyl chain protrudes straight toward TM5 and the benzyl group intercalates between TM1 and TM7 (Figure 6A). In particular, the ligand shows a π-π stacking with Y3.32(101) and hydrophobic interactions with M7.39(274) formed through its pyridone ring, while the fused phenyl ring forms extensive aromatic and lipophilic interactions with F3.33(102) and M3.36(105) (Figure 6B); interestingly, Y3.32(101) and M3.36(105) proved to be deeply involved in agonist signaling at the GPR55 receptor [30]. The methoxyl substituent of the ligand bicyclic core forms an H-bond with S7.42(277) that is maintained for most of the MD simulation, while the carbonyl group of the compound shows a less stable H-bond with K2.60(80). The ligand alkyl chain is placed in a hydrophobic region of the protein binding site delimited by F3.33(102), Y3.37(106), V6.51(242), H6.52(243) and F6.55(246) (this latter residue experimentally determined to be involved in agonist signaling), thus forming lipophilic interactions with all these residues. Finally, the benzyl group is sandwiched between Q1.35(23) and M7.39(274), forming hydrophobic interactions predominantly with these two residues and to a lesser extent with M1.31(19), H1.39(27) and C7.40(275), while the methoxy substituent seems not to form any particular interaction.

### 2.5. Pharmacokinetics Properties Prediction

In order to gain insight into the pharmacokinetic profile of the four synthesized compounds, two key ADME properties, i.e., blood–brain barrier (BBB) permeation and gastrointestinal absorption, were calculated by employing the web tools Online BBB Predictor and SwissADME. The ligands were predicted as BBB permeable with good confidence, since they were labelled as BBB-positive (BBB+), both by all eight predictive methods available in the Online BBB Predictor tool and by the BOILED-Egg method available in SwissADME. Furthermore, it was predicted that all four compounds would be well absorbed by the gastrointestinal tract, showing favorable LOGP and TPSA values (see the section “Materials and Methods” for details). Indeed, the calculated LOGP of the ligands did not exceed the threshold value of 5 included in Lipinski’s parameters and the value of TPSA was in the optimal range between 20 and 130 Å^2^. Therefore, it was predicted that the four compounds would have favorable pharmacokinetic properties.

## 3. Discussion

The new derivatives generally showed high levels of affinity towards GPR55. Compound **2**, bearing a methyl substituent on the nitrogen atom at position 1 of the central quinolin-2(1*H*)-one nucleus and two methoxyl groups at positions 5 and 2′, displayed the greatest affinity towards GPR55 within the series (*K*_i_ = 1.2 nM), showing also complete selectivity over CB1R (*K*_i_ > 10,000 nM) and a remarkable affinity for CB2R (*K*_i_ = 6.9 nM). For compound **1**, the removal of the methyl group from the nitrogen atom of compound **2** led to complete selectivity over CB1R and CB2R (*K*_i_ > 10,000 nM for both receptor), while maintaining a good affinity for GPR55 (*K*_i_ = 14 nM), although lower than compound **2**. The replacement of the two methoxyl groups of compound **2** with two hydroxyl groups, as in compound **4**, preserved a good affinity for GPR55 (*K*_i_ = 7.1 nM), but at the same time led to a complete loss of selectivity over both cannabinoid receptors (*K*_i_ CB1R = 29 nM; *K*_i_ CB2R = 7.9 nM). In the case of compound **3**, in which both hydroxyl groups are free and the nitrogen atom is demethylated, a high affinity for GPR55 (*K*_i_ = 6.2 nM) and CB1R (*K*_i_ = 5.1 nM) together with a complete lack of CB2R affinity (*K*_i_ > 10,000 nM) is observed. 

These data suggest that the methylation of the nitrogen atom does not greatly affect the affinity of these compounds towards the GPR55 receptor. Similarly, the substitution of the two methoxyl groups at positions 5 and 2′ of the quinolinone scaffold with two hydroxyl groups only slightly impacts the GPR55 affinity of the compounds. These structural features seem instead to play a crucial role in the interactions with both CBRs. In particular, *N*-methylation is fundamental for the affinity towards CB2R, while the presence of both free hydroxyl groups is important for the binding to CB1R. Therefore, the presence of two methoxyl groups at positions 5 and 2′, together with the removal of the methyl substituent from the nitrogen atom, as in compound **1**, allows for high GPR55 affinity and a complete selectivity over both cannabinoid receptors. For this reason, compound **1** is indeed the most interesting one within this series of new 3-benzylquinolin-2(1*H*)-ones.

As far as the pharmacological profile is concerned, all the newly synthesized compounds were full agonists of GPR55 relative to CP55,940 and O-1602, enhancing p-ERK1/2 levels in a dose-dependent manner and showing EC_50_ values in the low nanomolar range. In particular, derivatives **2** and **3** were the most potent agonists in the p-ERK assay. These compounds were similarly observed to have the greatest estimated affinity at GPR55. 

The molecular modeling study highlighted that the interactions observed in the predicted binding mode of **2** seem to be consistent with some of the preliminary above-mentioned SAR data obtained for this series of ligands. For instance, the role as a hydrogen bond acceptor of the methoxy substituent of the ligand core and the absence of relevant interactions formed by the methoxyl group on the benzyl ring may justify the only marginal decrease of activity, probably associated with desolvation effects, observed with replacement of both methoxyl groups by hydroxylic moieties, as in compound **4** (*K*_i_ = 7.1 nM).

## 4. Materials and Methods

### 4.1. Chemistry

#### 4.1.1. General Methods

Commercially available reagents were purchased from Sigma Aldrich, Zentek or Alfa Aesar and used without purification, if not differently indicated in the procedures. ^1^H NMR and ^13^C NMR spectra were recorded on a Bruker AVANCE III^TM^ 400 spectrometer (operating at 400 MHz or 100 MHz, respectively). Chemical shifts (*δ*) are reported in parts per million (ppm) related to the residual solvent signal, while coupling constants (*J*) are expressed in Hertz (Hz). All final compounds were analysed by HPLC, showing a purity in the range 95–99%. An HPLC instrument (Thermo Finnigan-Spectra System SCM1000) equipped with a Spectra System P2000 (Pumps), Spectra System UV2000, set to 280 nm was used. Analyses were achieved on Phenomenex Gemini reverse-phase C18 column (250 × 4.6 mm, 5 μm particle size; Phenomenex, Castel Maggiore, Italy). The mobile phase was constituted by a mixture of H_2_O/AcOH (0.1% *v*/*v*) (eluent A) and ACN (eluent B). An isocratic elution with 70% of B and 30% of A was chosen. The flow rate was 0.8 mL/min. Retention times are given in minutes. HRMS experiments were performed using a high-resolution mass spectrometer Orbitrap Q exactive plus (Thermo, San Jose, CA, USA) with HESI source. Spectra were recorded through direct injection at a flux of 5 (positive) and 7 (negative) μL min^−1^. Spectra were acquired with a nominal resolution of 140000 (*m*/*z* 200). Positive polarity: spray voltage 3.4 kV, capillary temperature 290 °C, S-lens RF level 50, sheath and auxiliary gas 24 and 5 (arbitrary units). Negative polarity: spray voltage −3.2 kV, capillary temperature 290 °C, S-lens RF level 50, sheath and auxiliary gas 28 and 4 (arbitrary units). Data elaboration: Xcalibur 4.2 (Thermo). Evaporation was carried out in vacuo using a rotating evaporator. Silica gel flash chromatography was performed using silica gel 60 Å (0.040–0.063 mm; Merck). Reactions were monitored by TLC on Merck aluminum silica gel (60 F254) plates that were visualized under a UV lamp (λ = 254 nm). Melting points were determined on a Kofler hot-stage apparatus and are uncorrected.

#### 4.1.2. 1-n-Butyl-3-methoxy-5-nitrobenzene (**6**)

Commercial 3-bromo-5-nitroanisole **5** (300.0 mg, 1.29 mmol) was dissolved in a mixture of toluene/H_2_O (5.0 mL/0.5 mL) in a vial under nitrogen flux. *n*-Butylboronic acid (158.0 mg, 1.55 mmol), *t-*BuONa (372.0 mg, 3.87 mmol), Pd_2_(dba)_3_ (24.0 mg, 0.03 mmol) and Ruphos (24.0 mg, 0.05 mmol) were sequentially added to the mixture at room temperature. The vial was sealed and left under magnetic stirring at 80 °C overnight. After cooling to room temperature, the reaction mixture was filtered over a silica pad and washed with AcOEt. The filtrate was evaporated in vacuo and the residue was purified by flash column chromatography on silica gel (*n*-hexane/AcOEt 9.5:0.5), affording intermediate **6** (210.0 mg, 1.00 mmol) as a transparent oil. Yield: 78%. ^1^H-NMR: (CDCl_3_) δ (ppm): 7.66–7.65 (m, 1H, ar); 7.55 (m, 1H, ar), 7.04–7.03 (m, 1H, ar), 3.87 (s, 3H, OCH_3_), 2.66 (t, 2H, *J* = 7.7 Hz, C*H_2_*CH_2_CH_2_CH_3_), 1.66–1.58 (m, 2H, CH_2_C*H_2_*CH_2_CH_3_), 1.41–1.32 (m, 2H, CH_2_CH_2_C*H_2_*CH_3_), 0.93 (t, 3H, *J* = 7.3 Hz, CH_2_CH_2_CH_2_C*H_3_*). ^13^C-NMR (CDCl_3_) δ (ppm): 160.14 (*C*-OCH_3_), 149.35 (*C*-NO_2_), 145.88 (*C*-*n*-Bu), 121.60 (C6), 116.01 (C2), 105.52 (C4), 55.91 (O*C*H_3_), 35.57 (*C*H_2_CH_2_CH_2_CH_3_), 33.22 (CH_2_*C*H_2_CH_2_CH_3_), 22.34 (CH_2_CH_2_*C*H_2_CH_3_), 13.99 (CH_2_CH_2_CH_2_*C*H_3_). 

#### 4.1.3. 3-n-Butyl-5-methoxyaniline (**7**)

Intermediate **6** (710.0 mg, 3.39 mmol) was dissolved in a mixture of EtOH/H_2_O (42.0 mL/21.0 mL). After the addition of iron powder (2.20 g, 39.39 mmol) and NH_4_Cl (1.05 g, 19.56 mmol), the reaction mixture was refluxed for 3 h. After cooling to room temperature, the reaction mixture was filtered over a celite pad and washed with EtOH. The filtrate was evaporated in vacuo and the residue was dissolved in AcOEt and washed with distilled H_2_O. The organic phase was dried over anhydrous Na_2_SO_4_, filtered and evaporated, affording compound **7** (540.0 mg, 3.01 mmol) as an orange oil, which was used for the next step without further purification. Yield: 89%. ^1^H-NMR (CDCl_3_) δ (ppm): 6.19–6.16 (m, 2H, ar), 6.10 (m, 1H, ar), 3.75 (s, 3H, OCH_3_), 2.48 (t, 2H, *J* = 7.6 Hz, C*H_2_*CH_2_CH_2_CH_3_), 1.60–1.53 (m, 2H, CH_2_C*H_2_*CH_2_CH_3_), 1.37–1.32 (m, 2H, CH_2_CH_2_C*H_2_*CH_3_), 0.92 (t, 3H, *J* = 7.3 Hz, CH_2_CH_2_CH_2_C*H_3_*). ^13^C-NMR (CDCl_3_) δ (ppm): 160.84 (=*C*-OCH_3_), 147.56 (=*C*-NH_2_), 145.59 (C5), 108.31 (C2), 104.72 (C4), 98.59 (C6), 55.18 (O*C*H_3_), 35.96 (*C*H_2_CH_2_CH_2_CH_3_), 33.51 (CH_2_*C*H_2_CH_2_CH_3_), 22.55 (CH_2_CH_2_*C*H_2_CH_3_), 14.09 (CH_2_CH_2_CH_2_*C*H_3_).

#### 4.1.4. Methyl 3-(2-hydroxyphenyl)propanoate (**9**)

Concentrated H_2_SO_4_ (0.3 mL) was added to a solution of commercial 3,4-dihydrocumarine **8** (2.00 g, 13.5 mmol) in anhydrous MeOH (15.0 mL) under nitrogen atmosphere, and the resulting solution was refluxed overnight. Ambersep 900 OH resin was added until approximately pH = 7.0 was reached and then the solution was filtered and washed with AcOEt. The filtrate was evaporated in vacuo and the residue was dissolved in AcOEt and washed with distilled H_2_O and brine. The organic phase was dried over anhydrous Na_2_SO_4_, filtered and evaporated, affording intermediate **9** (2.14 g, 11.9 mmol) as a yellow oil, which was used for the next step without further purification. Yield: 88%. ^1^H-NMR (CDCl_3_) δ (ppm): 7.14–7.08 (m, 2H, ar), 7.07 (bs, 1H, OH), 6.89–6.85 (m, 2H, ar), 3.69 (s, 3H, OCH_3_), 2.91 (t, 2H, *J* = 6.8 Hz, C*H_2_*CH_2_(CO)), 2.72 (t, 2H, *J* = 7.2 Hz, CH_2_C*H_2_*(CO)). ^13^C-NMR (CDCl_3_) δ (ppm): 175.98 (*C*=O), 154.27 (*C*-OH), 130.55 (C6′), 128.03 (C5′), 127.26 (C4′), 120.88 (C1′), 116.94 (C3′), 52.28 (O*C*H_3_), 34.90 (CH_2_*C*H_2_COOH), 24.98 (*C*H_2_CH_2_COOH).

#### 4.1.5. Methyl 3-(2-methoxyphenyl)propanoate (**10**)

Intermediate **9** (3.14 g, 17.4 mmol) was added to a suspension of NaH (60% dispersion in mineral oil) (1.44 g) in DMF (10 mL) at 0 °C. The suspension was stirred at room temperature. After 30 min, CH_3_I (2.17 mL, 34.8 mmol) was added dropwise at 0 °C and then the reaction mixture was stirred at room temperature overnight. After the addition of about 10 mL of distilled H_2_O, the aqueous phase was extracted with AcOEt. After washing with distilled H_2_O, the organic phase was dried over anhydrous Na_2_SO_4_, filtered and evaporated, affording compound **10** (0.80 g, 4.07 mmol) as a transparent oil, which was used for the next step without further purification. Yield: 92%. ^1^H-NMR (CDCl_3_) δ (ppm): 7.22–7.13 (m, 2H, ar), 6.89–6.83 (m, 2H, ar), 3.96 (s, 3H, OCH_3_), 3.66 (s, 3H, OCH_3_), 2.94 (t, 2H, *J* = 7.6 Hz, C*H_2_*CH_2_(CO)), 2.61 (t, 2H, *J* = 7.6 Hz, CH_2_C*H_2_*(CO)). ^13^C-NMR (CDCl_3_) δ (ppm): 173.99 (*C*=O), 157.62 (=*C*-OCH_3_), 130.05 (C6′), 128.96 (C4′), 127.76 (C5′), 120.57 (C1′), 110.35 (C3′), 55.32 (C2′-O*C*H_3_), 51.63 (COO*C*H_3_), 34.17 (CH_2_*C*H_2_(CO)), 26.27 (*C*H_2_CH_2_ (CO)).

#### 4.1.6. 3-(2-Methoxyphenyl)propanoic Acid (**11**)

LiOH (0.52 g, 21.76 mmol) was added to a solution of intermediate **10** in MeOH/H_2_O (16.0 mL/5.0 mL) and the reaction mixture was stirred at room temperature for 3 h. MeOH was evaporated in vacuo and the residue was diluted with distilled H_2_O and washed with AcOEt. The aqueous phase was acidified with 1 M HCl until approximately pH = 4 was reached, and subsequently extracted with AcOEt. The organic phase was dried over anhydrous Na_2_SO_4_, filtered and evaporated, affording derivative **11** (0.52 g, 2.88 mmol) as a white solid, which was used for the next step without further purification. Yield: 71%. ^1^H-NMR (CDCl_3_) δ (ppm): 7.23–7.15 (m, 2H, ar), 6.90–6.84 (m, 2H, ar), 3.82 (s, 3H, OCH_3_), 2.95 (t, 2H, *J* = 7.6 Hz, C*H_2_*CH_2_COOH), 2.61 (t, 2H, *J* = 7.6 Hz, CH_2_C*H_2_*COOH). ^13^C-NMR (CDCl_3_) δ (ppm): 180.06 (*C*=O), 157.59 (=*C*-OCH_3_), 130.05 (C6′), 128.60 (C1′), 127.83 (C5′), 120.58 (C4′), 110.33 (C3′), 55.26 (O*C*H_3_), 34.12 (CH_2_*C*H_2_COOH), 25.99 (*C*H_2_CH_2_COOH).

#### 4.1.7. *N*-(3-n-Butyl-5-methoxyphenyl)-3-(2-methoxyphenyl)propanamide (**13**)

Intermediate **11** (300.0 mg, 1.65 mmol) was dissolved in toluene (4.0 mL) in a vial, under nitrogen flux. SOCl_2_ (0.1 mL, 1.10 mmol) was added at room temperature and the vial was sealed and left under magnetic stirring at reflux for 3 h to allow the formation of the acyl chloride **12**. The solvent and the excess of unreacted SOCl_2_ were then removed under nitrogen flux. In a round bottom flask, under nitrogen atmosphere, Et_3_N (0.1 mL, 0.745 mmol) was added to a solution of compound **7** (270.0 mg, 1.25 mmol) in DCM (10.0 mL). The acyl chloride **12**, previously dissolved in DCM (10.0 mL), was then added dropwise and the reaction was stirred at room temperature overnight. The resulting solution was diluted with DCM and washed with an aqueous solution of NaOH (10% _m/v_), 1 M HCl and distilled H_2_O. The organic phase was dried over anhydrous Na_2_SO_4_, filtered and evaporated, affording compound **13** (370.0 mg, 1.26 mmol) as a light-brown solid, which was used for the next step without further purification. Yield > 99%. ^1^H-NMR (CDCl_3_) δ (ppm): 7.24–7.18 (m, 2H, ar), 7.06–7.03 (m, 2H, ar), 6.91–6.86 (m, 2H, ar), 6.74 (s, 1H, ar), 6.48 (bs, 1H, NH), 3.84 (s, 3H, OCH_3_), 3.78 (s, 3H, OCH_3_), 3.04 (t, 2H, *J* = 7.3 Hz, (C=O)CH_2_C*H_2_*), 2.63 (t, 2H, *J* = 7.8 Hz, (C=O)C*H_2_*CH_2_), 2.53 (t, 2H, *J* = 7.8 Hz, C*H_2_*CH_2_CH_2_CH_3_), 1.57–1.53 (m, 2H, CH_2_C*H_2_*CH_2_CH_3_), 1.36–1.31 (m, 2H, CH_2_CH_2_C*H_2_*CH_3_), 0.91 (t, 3H, *J* = 7.3 Hz, CH_2_CH_2_CH_2_C*H_3_*). ^13^C-NMR (CDCl_3_) δ (ppm): 170.86 (*C*=O), 160.02 (C2”), 157.33 (C5′), 144.92 (C1′), 138.86 (C3′), 130.21 (C4”), 128.83 (C6”), 127.76 (C5”), 120.71 (C1”), 112.01 (C2′), 110.41 (C3”), 110.37 (C4′), 102.61 (C6′), 55.31 (C2”), 55.27 (C5′), 38.03 (*C*H_2_CH_2_CH_2_CH_3_), 35.74 (CH_2_*C*H_2_CH_2_CH_3_), 33.37 (CH_2_*C*H_2_(CO)), 26.54 (*C*H_2_CH_2_(CO)), 22.33 (CH_2_CH_2_*C*H_2_CH_3_), 13.95 (CH_2_CH_2_CH_2_*C*H_3_).

#### 4.1.8. 7-n-Butyl-2-chloro-5-methoxy-3-(2-methoxybenzyl)quinoline (**14**)

Intermediate **13** (200.0 mg, 0.59 mmol) was dissolved in anhydrous DMF (0.06 mL, 0.71 mmol) in a vial. POCl_3_ (0.4 mL, 4.13 mmol) was added at 10 °C under nitrogen flux and the vial was sealed and left under magnetic stirring at 80 °C overnight. The reaction mixture was poured into ice and H_2_O, diluted with H_2_O and extracted with DCM. The organic phase was dried over anhydrous Na_2_SO_4_, filtered, evaporated and the residue was purified with flash column chromatography on silica gel (*n*-hexane/AcOEt 9.5:0.5), affording compound **14** (70.0 mg, 0.19 mmol) as a white solid. These conditions allowed the effective separation of **14** from the positional isomer **15** (see Supplementary Materials, Figure S2). Yield: 32%. M. p.: 82–84 °C. ^1^H-NMR (CDCl_3_) δ (ppm): 8.19 (s, 1H, H4); 7.38 (s, 1H, H6′); 7.26–7.25 (m, 1H, H5′), 7.06–7.05 (m, 1H, H3′), 6.91–6.89 (m, 2H, H8, H4′), 6.65 (s, 1H, H6), 4.19 (s, 2H, benzylic), 3.92 (s, 3H, C2′-OC*H_3_*), 3.82 (s, 3H, C5-OC*H_3_*), 2.76 (t, 2H, *J* = 7.6 Hz, C*H_2_*CH_2_CH_2_CH_3_), 1.70–1.66 (m, 2H, CH_2_C*H_2_*CH_2_CH_3_), 1.41–1.36 (m, 2H, CH_2_CH_2_C*H_2_*CH_3_), 0.92 (t, 3H, *J* = 7.2 Hz, CH_2_CH_2_CH_2_C*H_3_*). ^13^C-NMR (CDCl_3_) δ (ppm): 157.49 (C2′), 154.62 (C5), 152.24 (C2), 147.58 (C10), 145.30 (C7), 133.30 (C6′), 130.59 (C11), 130.30 (C3), 127.91 (C4′), 127.14 (C1′), 120.53 (C8), 118.83 (C5′), 118.38 (C9), 110.44 (C3′), 106.19 (C6), 55.55 (C5-O*C*H_3_), 55.32 (C2′-O*C*H_3_), 36.38 (C1”), 33.52 (C2”), 33.01 (C11), 22.28 (C3”), 13.95 (C4”). HRMS (HESI): *m/z* calculated for C_22_H_24_ClNO_2_ + H^+^ [M + H]^+^: 370.15695. Found: 370.15683. HPLC analysis: retention time = 16.65 min; peak area, 97.1% (280 nm).

#### 4.1.9. 7-n-Butyl-5-methoxy-3-(2-methoxybenzyl)quinolin-2(1H)-one (**1**)

Compound **14** (200.0 mg, 0.54 mmol) was suspended in HCl 6 N (5.5 mL) in a vial. The vial was then sealed and heated at reflux overnight. The reaction mixture was diluted with distilled H_2_O and extracted with DCM. The organic phase was dried over anhydrous Na_2_SO_4_, filtered and evaporated. The residue was purified with flash column chromatography on silica gel (*n*-hexane/AcOEt 7:3), affording compound **1** (100.0 mg, 0.28 mmol), as a white solid. Yield: 53%. M. p.: 208–210 °C. ^1^H-NMR (CDCl_3_) δ (ppm): 11.29 (bs, 1H, exchangeable, N*H*), 7.82 (s, 1H, H4), 7.30–7.28 (m, 1H, H6′), 7.24–7.19 (m, 1H, H5′), 6.92–6.88 (m, 2H, H3′, H4′), 6.65 (s, 1H, H8), 6.40 (s, 1H, H6), 4.00 (s, 2H, benzylic), 3.86 (s, 3H, C2′-OC*H_3_*), 3.82 (s, 3H, C5-OC*H_3_*), 2.65 (t, 2H, *J* = 7.6 Hz, CH_2_), 1.66–1.59 (m, 2H, CH_2_C*H_2_*CH_2_CH_3_), 1.42–1.35 (m, 2H, CH_2_CH_2_C*H_2_*CH_3_), 0.92 (t, 3H, *J* = 7.2 Hz, CH_2_CH_2_CH_2_C*H_3_*). ^13^C-NMR (CDCl_3_) δ (ppm): 157.72 (C2), 155.46 (C5, C2′), 145.99 (C7), 138.57 (C10), 132.21 (C6′), 130.89 (C4), 127.95 (C3), 127.51 (C1′, C4′), 120.49 (C8), 110.52 (C5′), 109.33 (C9), 107.43 (C3′), 103.45 (C6), 55.55 (C5-O*C*H_3_), 55.40 (C2′-O*C*H_3_), 36.40 (C1”), 33.42 (C11), 30.37 (C2”), 22.46 (C3”), 13.96 (C4”). HRMS (HESI): *m/z* calculated for C_22_H_25_NO_3_ + H^+^ [M + H]^+^: 352.19084. Found: 352.19072. HPLC analysis: retention time = 11.01 min; peak area, 95.2% (280 nm).

#### 4.1.10. 7-n-Butyl-5-methoxy-3-(2-methoxybenzyl)-1-methylquinolin-2(1H)-one (**2**)

To a solution of compound **1** (0.11 g, 0.31 mmol) in anhydrous DMF (2.0 mL), under nitrogen atmosphere, NaH (60% dispersion in mineral oil) (25.0 mg, 1.04 mmol) was added. Upon the completion of gas evolution, CH_3_I (0.03 mL, 0.38 mmol) was added and the solution was stirred at room temperature overnight. The reaction mixture was diluted with distilled H_2_O to remove the excess of NaH, and extracted with DCM. The organic phase was dried over anhydrous Na_2_SO_4_, filtered and evaporated. The residue was purified by flash column chromatography on silica gel (*n*-hexane/AcOEt 7:3), affording compound **2** (60.0 mg, 0.16 mmol) as a light yellow solid. Yield: 53%. M. p.: 78–80 °C. ^1^H-NMR (CDCl_3_) δ (ppm): 7.80 (s, 1H, H4), 7.26–7.19 (m, 2H, H5′, H6′), 6.92–6.87 (m, 2H, H3′, H4′), 6.73 (s, 1H, H8), 6.47 (s, 1H, H6), 3.97 (s, 2H, benzylic), 3.87 (s, 3H, C2′-OC*H_3_*), 3.81 (s, 3H, C5-OC*H_3_*), 3.72 (s, 3H, N-C*H_3_*), 2.65 (t, 2H, *J* = 7.6 Hz, C*H_2_*CH_2_CH_2_CH_3_), 1.68–1.61 (m, 2H, CH_2_C*H_2_*CH_2_CH_3_), 1.44–1.34 (m, 2H, CH_2_CH_2_C*H_2_*CH_3_), 0.95 (t, 3H, *J* = 7.2 Hz, CH_2_CH_2_CH_2_C*H_3_*). ^13^C-NMR (CDCl_3_) δ (ppm): 162.75 (C2), 157.73 (C2′), 155.87 (C5), 145.75 (C7), 140.22 (C10), 130.84 (C6′), 130.03 (C4), 129.58 (C3), 127.97 (C4′), 127.46 (C1′), 120.51 (C8), 110.53 (C5′), 109.38 (C9), 106.29 (C3′), 103.41 (C6), 55.69 (C5-O*C*H_3_), 55.40 (C2′-O*C*H_3_), 36.93 (C1”), 33.69 (C11), 31.34 (C2”), 30.08 (N-*C*H_3_), 22.47 (C3”), 13.97 (C4”). HRMS (HESI): *m/z* calculated for C_23_H_27_NO_3_ + H^+^ [M + H]^+^: 366.20649. Found: 366.20637. HPLC analysis: retention time = 18.94 min; peak area, 97.7% (280 nm).

#### 4.1.11. General Procedure for the Synthesis of Compounds **3** and **4**

BBr_3_ (1 M solution in DCM) (3 eq) was added dropwise, under nitrogen flux, to a solution of compound **1** or **2** (100.0 mg, 0.28 mmol) in DCM (5.0 mL), previously cooled at −78 °C. The cold bath was then removed and the reaction mixture was stirred at room temperature overnight. The solution was quenched with distilled H_2_O (10.0 mL) and the organic and aqueous phases were separated. The aqueous phase was extracted with AcOEt and the combined organic layers were collected, dried with anhydrous Na_2_SO_4_, filtered and evaporated.

7-*n*-Butyl-5-hydroxy-3-(2-hydroxybenzyl)quinolin-2(1*H*)-one (**3**)

The residue was purified with flash column chromatography on silica gel (*n*-hexane/AcOEt 7:3), affording **3** (60.0 mg, 0.18 mmol) as a white solid. Yield: 66%. M. p.: 252–254 °C. ^1^H-NMR (DMSO-*d_6_*) δ (ppm): 11.72 (bs, 1H, exchangeable, N*H*), 10.11 (bs, 1H, exchangeable, C5-O*H*), 9.63 (bs, 1H, exchangeable, C2′-O*H*), 7.71 (s, 1H, H4), 7.14 (dd, 1H, *J* = 7.6 Hz, *J* = 1.2 Hz, H6′), 7.07–7.03 (m, 1H, H5′), 6.80 (d, 1H, *J* = 7.2 Hz, H3′), 6.74 (t, 1H, *J* = 7.2 Hz, H4′), 6.41 (s, 1H, H8), 6.40 (s, 1H, H6), 3.72 (s, 2H, benzylic), 2.54–2–48 (m, 2H, C*H_2_*CH_2_CH_2_CH_3_), 1.54–1.47 (m, 2H, CH_2_C*H_2_*CH_2_CH_3_), 1.33–1.23 (m, 2H, CH_2_CH_2_C*H_2_*CH_3_), 0.88 (t, 3H, *J* = 7.6 Hz, CH_2_CH_2_CH_2_C*H_3_*). ^13^C-NMR: (DMSO-*d_6_*) δ (ppm): 163.37 (C2), 155.74 (C2′), 153.90 (C5), 145.69 (C7), 139.41 (C10), 131.61 (C6′), 131.17 (C4), 129.71 (C3), 127.94 (C4′), 126.44 (C1′), 119.63 (C8), 116.14 (C5′), 108.09 (C3′), 107.62 (C6), 105.63 (C9), 35.66 (C1”), 33.20 (C11), 30.70 (C2”), 22.17 (C3”), 14.25 (C4”). HRMS (HESI): *m/z* calculated for C_20_H_21_NO_3_ + H^+^ [M + H]^+^: 324.15953. Found: 324.15942. HPLC analysis: retention time = 5.49 min; peak area, 95.2% (280 nm).

7-*n*-Butyl-5-hydroxy-3-(2-hydroxybenzyl)-1-methylquinolin-2(1*H*)-one (**4**)

The residue was purified with flash column chromatography on silica gel (*n*-hexane/AcOEt/MeOH 9:0.8:0.2), affording **4** (20 mg, 0.06 mmol) as a white solid. Yield: 42%. M. p.: 223–225 °C. ^1^H-NMR (acetone-*d_6_*) δ (ppm): 9.97 (bs, 1H, exchangeable, C5-O*H*), 9.29 (bs, 1H, exchangeable, C2′-O*H*), 8.31 (s, 1H, H4), 7.34 (dd, 1H, *J* = 7.2 Hz, *J* = 1.6 Hz, H6′), 7.07 (dt, 1H, *J* = 7.2 Hz, *J* = 1.6 Hz, H5′), 6.93 (s, 1H, H8), 6.93–6.71 (m, 2H, H3′, H4′), 6.70 (s, 1H, H6), 3.93 (s, 2H, benzylic), 3.75 (s, 3H, N-C*H_3_*), 2.68 (t, 2H, *J* = 7.6 Hz, C*H_2_*CH_2_CH_2_CH_3_), 1.66–1.60 (m, 2H, CH_2_C*H_2_*CH_2_CH_3_), 1.40–1.35 (m, 2H, CH_2_CH_2_C*H_2_*CH_3_), 0.91 (t, 3H, *J* = 7.2 Hz, CH_2_CH_2_CH_2_C*H_3_*). ^13^C-NMR: (acetone-*d_6_*) δ (ppm): 164.68 (C2), 156.62 (C5), 155.02 (C2′), 147.84 (C7), 141.08 (C10), 132.32 (C6′), 131.58 (C4), 129.75 (C3), 128.68 (C4′), 127.80 (C1′), 120.65 (C8), 118.20 (C5′), 109.83 (C9), 109.21 (C3′), 106.65 (C6), 36.97 (C1”), 34.22 (C11), 33.23 (C2”), 30.07 (N-*C*H_3_), 22.99 (C3”), 14.15 (C4”). HRMS (HESI): *m/z* calculated for C_21_H_23_NO_3_ + H^+^ [M + H]^+^: 338.17519. Found: 338.17507. HPLC analysis: retention time = 8.24 min; peak area, 96.1% (280 nm).

### 4.2. Biological Evaluation

#### 4.2.1. Compounds

CP55,940 and O-1602 were purchased from Cayman Chemicals (Ann Arbor, MI, USA). [^3^H]CP55,940 (174.6 Ci/mmol) was obtained from PerkinElmer (Guelph, ON, Canada). Unless stated, all other reagents were obtained from Sigma-Aldrich (Oakville, ON, Canada). Compounds were dissolved in DMSO (final concentration of 0.1% in assay media for all assays) and added directly to the media at the concentrations and times indicated.

#### 4.2.2. Cell Culture

Chinese hamster ovary (CHO)-K1 cells, either untransfected or stably expressing *h*CB1R, or *h*CB2R, have been described in earlier studies from our group [27,31]. CHO-K1 cells were originally sourced from ATCC (Catalog No. CCL-61; Manas-sas, VI). CHO-K1 *h*CB1R and *h*CB2R cells were originally sourced from Eurofins (DiscoveRx; HitHunter^®^, Catalog Nos. 95-0071C2 and 95-0183C2, respectively; Fremont, CA, USA). CHO-K1 cells expressing *h*GPR55 were produced by transfecting CHO-K1 cells with 2 µg pcDNA3.1+-*h*GPR55 plasmid (cDNA Resource Centre, Bloomsburg, PA; Cat No. GPR0550000) according to the Lipofectamine 3000 standard operating procedure (ThermoFisher Scientific, Waltham, MA, USA). Cells were maintained at 37°C, 5% CO_2_ in F-12/DMEM containing 1 mM l-glutamine, 10% FBS and 1% Pen/Strep; hygromycin B (300 µg/mL) and G418 (600 µg/mL) were used for CHO *h*CB1R cells, and G418 (400 µg/mL) was used for CHO *h*CB2R cells. For the collection of membranes to be used in radioligand displacement assays, cells were grown in 15 cm^2^ dishes to confluency, washed in 0.1 M PBS, scraped from dishes, centrifuged and frozen at −80 °C until required. 

#### 4.2.3. [^3^H]CP55,940 Radioligand Displacement Assay

Assays were performed as described previously and summarized here [27]. Assays used 1 nM [^3^H]CP55,940 and Tris binding buffer (50 mM Tris−HCl, 50 mM Tris−base, 0.1% BSA, pH 7.4), in a total assay volume of 500 μL. Binding was initiated by the addition of transfected *h*GPR55, *h*CB1R or *h*CB2R CHO cell membranes (25 μg protein per well). All assays were performed at 37°C for 120 min prior to quenching with ice-cold Tris binding buffer followed by vacuum filtration using a 24-well sampling manifold (Brandel Cell Harvester; Brandel, Inc., Gaithersburg, MD, USA) and Brandel GF/B filters. Each reaction well was washed six times with 1.2 mL aliquots of Tris-binding buffer. The filters were air-dried overnight and then placed in 5 mL of scintillation fluid (Ultima Gold XR, Perkin Elmer, Inc., Waltham, MA, USA). Liquid scintillation spectrometry was used for quantifying radioactivity. Specific binding was defined as the difference between the binding that occurred in the presence and absence of 1 μM unlabelled CP55,940. Data are presented as % [^3^H]CP55,940 bound.

#### 4.2.4. In-Cell Western Quantification of ERK1/2 Phosphorylation

Cells were seeded at approximately 2000 cells/well in 96-well plates and treated with 0.1–10 μM compounds for 30 min and were then fixed for 10 min at room temperature with 4% paraformaldehyde. Cells were washed 3 times with 100 μL 1X PBS for 5 min each. Cells were incubated with blocking solution (PBS, 20% Odyssey blocking buffer, and 0.1% TritonX-100) for 1 h at room temperature. Next, cells were exposed to primary antibodies for p-ERK1/2(T202/Y204) (mouse monoclonal IgG, Cat# sc-136521) or ERK1/2 (rabbit polyclonal IgG, Cat# sc-292838) (Santa Cruz Biotechnology, Santa Cruz, CA) diluted (1:200) in blocking solution overnight at 4 °C. Cells were washed 3 times with 100 μL PBS for 5 min each. Cells were exposed to the secondary antibodies goat polyclonal anti-rabbit IgG IR^Dye800^-conjugated antibody diluted 1:500 (Li-Cor Biosciences, IgG, Cat# P/S 926-32210, RRID AB-2687825, lot# C50113-18) and goat polyclonal anti-mouse IgG IR^Dye680^-conjugated antibody diluted 1:500 (Li-Cor Biosciences, IgG, Cat# P/N 925-68070, RRID AB-2651128, lot# C50113-17) for 1 h at room temperature. Cells were washed 3 times with 100 μL PBS for 5 min each. Analyses were conducted using the Odyssey Imaging system and software (version 3.0; Li-Cor).

#### 4.2.5. Statistical Analyses

[^3^H]CP55,940 radioligand competition binding data are provided as % maximal ^3^H bound (i.e., 100%). Data for p-ERK in-cell westerns are shown as fold over vehicle response (i.e., 1). Estimates of *K*_i_, EC_50_ and *E*_max_ were determined using non-linear regression (3 parameters) (GraphPad, Prism, v. 9.0). *p* < 0.05 was determined to be significant. Values are presented as the mean ± the standard error of the mean (SEM) or 95% confidence interval (CI), as indicated in tables and figure legends.

### 4.3. Molecular Modeling

#### 4.3.1. Homology Modeling and Protein Structure Refinement

Homology modeling studies were performed using a model of *h*GPR55 generated based on the X-ray structure of zebrafish lysophosphatidic acid receptor LPA_6_ in complex with LPA, its endogenous agonist ligand (PDB code 5XSZ) [28]. The template for homology modeling was selected based on a sequence alignment performed with Protein Blast [32], highlighting zebrafish LPA_6_ receptor as the protein with the highest level of sequence homology to *h*GPR55 among those included in the Protein Data Bank, whose structure was experimentally determined. Moreover, the ligand co-crystallized with the receptor in the X-ray structure of LPA_6_ selected as a template is structurally related to the endogenous agonists of *h*GPR55 [33]. The *h*GPR55 homology model was then generated using Modeller software [34]. A total of 100 different models were generated and the model presenting the best value DOPE score was then selected for structural refinement through molecular dynamics (MD) simulations.

#### 4.3.2. hGPR55 Model Refinement

Prior to MD simulations, the eight and six residues at the extremities of the protein N-terminal and C-terminal loops, respectively, were removed from the model. The protein was then embedded in a lipid bilayer composed of POPC (1-palmitoyl-2-oleoyl-sn-glycero-3-phosphocholine) molecules and subjected to energy minimization in an explicit water environment. The generation of the phospholipid bilayer and the insertion of the receptor within it were performed using Visual Molecular Dynamics (VMD) software [35]. The MD simulations were carried out with AMBER software, version 20 [36], using the ff14SB force field for the protein and Lipid17 for POPC molecules. The system was solvated with a 15 Å water cap on both the “intracellular” and the “extracellular” sides using the TIP3P solvent model, while chloride ions were added as counterions to neutralize the system. The obtained system was then subjected to energy minimizations and MD simulations adapted from a protocol successfully applied for studying transmembrane receptors [37,38]. Three sequential minimization stages, each consisting of 5000 steps of steepest descent followed by conjugate gradient, were performed. In the first stage, a position restraint of 100 kcal/mol·Å^2^ was applied to the whole protein and phospholipid bilayer in order to uniquely minimize the positions of the water molecules. In the second stage, the same position restraint was only applied to the protein residues, thus leaving the phospholipid molecules free, while in the last stage only the protein α-carbons were restrained with a harmonic potential of 30 kcal/mol·Å^2^. The minimized system was then used as input for the MD simulation, which were performed using particle mesh Ewald electrostatics with a cutoff of 10 Å for non-bonded interactions and periodic boundary conditions. A simulation step of 2.0 fs was employed, as all bonds involving hydrogen atoms were kept rigid using the SHAKE algorithm. Initially, the temperature of the system was gradually raised from 0 to 300 K through a brief constant-volume MD simulation where a position restraint of 30 kcal/mol·Å^2^ was still applied on the protein α-carbons. The system was then relaxed through a 500 ps constant-pressure MD simulation in which the harmonic potential applied to the protein α-carbons was decreased to 10 kcal/mol·Å^2^ and a Langevin thermostat was used to maintain the temperature at 300 K. Subsequently, a long equilibration stage consisting of 30 ns of constant-pressure MD simulation was performed using the Monte Carlo barostat with anisotropic pressure scaling. In this stage, the α-carbon restraint (10 kcal/mol·Å^2^) was maintained on the whole receptor. Finally, 200 ns of MD simulation production was performed using the same pressure and temperature control settings, but applying a harmonic restraint of 10 kcal/mol·Å^2^ only on the α-carbons of the protein α-helices, thus leaving all loop sequences free to move. The average structure, obtained using the Cpptraj program implemented in AMBER suite, from the last 80 ns of MD was used as the final *h*GPR55 model for further studies.

#### 4.3.3. Induced-Fit Docking

Molecular docking calculations were performed using AUTODOCK 4.2 software [29]. Maestro [39] was employed to build the ligand, while Macromodel [40] was used for its minimization in a water environment performed with the CG method until a convergence value of 0.05 kcal/Å·mol, MMFF force field and a distance-dependent dielectric constant of 1.0. Autodock Tools was then used to identify the torsion angles in the ligand, add the solvent model and calculate protein and ligand atomic charges. Kollmann charges were assigned to the protein and Gasteiger charges to the ligand. The grid used for docking calculations was enclosed in a box of 56, 56 and 50 points in the x, y and z directions, respectively, centered on the receptor binding pocket defined by Y3.32(101), M3.36(105), F6.48(239) and F6.55(246), which were found to be involved in agonist signaling at the GPR55 receptor [30]. A grid spacing of 0.375 Å and a distance-dependent function of the dielectric constant were used for the energetic map calculations. The ligand was subjected to a robust docking procedure with flexible sidechains, in which the sidechains of few protein residues within the binding pocket were allowed to move during docking [41]. In detail, the key residues Y3.32(101), M3.36(105), F6.48(239) and F6.55(246), as well as F3.33(102) and M7.39(274), which were found to partially occlude the entrance of the receptor binding pocket in the refined *h*GPR55 structure, were treated as flexible in the docking calculations, which were performed by applying 200 runs of Autodock search, using the Lamarckian Genetic Algorithm with 10,000,000 steps of energy evaluations. The number of individuals in the initial population was set to 500 and a maximum of 10,000,000 generations were simulated during each docking run. Cluster analysis was performed on the results using an RMS tolerance of 2.0 Å. The eight clusters of solutions with a population higher than 5%, i.e., including more than 5% of all the generated docking poses, were taken into account.

#### 4.3.4. Molecular Dynamics Simulations of Ligand-GPR55 Complexes

All MD simulations performed on the ligand–protein complexes generated through docking calculations were performed with AMBER 20, using the ff14SB force field for the protein, Lipid17 for POPC molecules and General Amber force field (GAFF) for the ligand. The MD protocol followed was comparable to that employed for the structural refinement of the *h*GPR55 model. Each complex was embedded in a lipid bilayer composed of POPC molecules, solvated with a 15 Å water cap on both the “intracellular” and the “extracellular” sides and subjected to the three stages of energy minimization described above. The minimized systems were then gradually heated to 300 K through a brief constant-volume MD stage and then relaxed through a 500 ps constant-pressure MD stage, applying the same harmonic restraints to all protein α-carbons (of 30 and 10 kcal/mol·Å^2^ during the first and second simulation stages, respectively), employed during *h*GPR55 model refinement. Finally, a 100 ns production stage of constant-pressure MD simulation was performed maintaining the position restraint of 10 kcal/mol·Å^2^ on all protein α-carbons. All other simulation parameters were set as for *h*GPR55 model refinement. The MD analysis was performed using the Cpptraj program implemented in AMBER suite.

#### 4.3.5. Binding Free Energy Evaluations

The ligand–protein binding affinity of the ligand-*h*GPR55 complexes analyzed through MD simulations were calculated with AMBER 20 using the Molecular Mechanics–Poisson–Boltzmann Surface Area (MM-PBSA) method, as previously performed [42,43]. The trajectories corresponding to the last 50 ns of MD simulation were used for the evaluation, which was performed on a total of 500 MD frames (one every 100 ps). The MOLSURF program and the MM-PBSA module of AMBER 20 were used to calculate nonpolar and polar energies, respectively, while the SANDER module estimated Waals, electrostatic and internal contributions. The ligand’s entropy was not taken into account in the calculation.

#### 4.3.6. Amino Acid Numbering

In referring to specific amino acids, both the sequence number (in parentheses) and the numbering system proposed by Ballesteros and Weinstein were employed [44]. For the latter, the most highly conserved residue in each TM helix (TMH) was assigned a value of 0.50, and this number was preceded by the TMH number. The other residues in the helix were given a locant value relative to this.

#### 4.3.7. Pharmacokinetics Properties Predictions

The blood–brain barrier (BBB) permeation and gastrointestinal absorption of the ligands were predicted using two online tools, BBB Online Predictor [45] and SwissADME [46]. The former is specifically designed to predict whether a compound can cross the blood–brain barrier (BBB+) or not (BBB-). In this work, we used AdaBoost and SVM models as classification algorithms, and MACCS, Openbabel FP2, Molprint 2D and PubChem fingerprints to represent the ligand structures, for a total of eight different combinations of predictive methods. SwissADME was used to evaluate both brain penetration (BBB) and gastrointestinal absorption by employing the BOILED-Egg method (brain or intestinal estimated permeation method), a predictive model that takes into account the lipophilicity and polarity of small molecules based on their predicted WLOGP and TPSA values [47].

## 5. Conclusions

In this work we reported a novel series of variously substituted 3-benzylquinolin-2(1*H*)-ones, designed and synthesized by applying some structural modifications to 3-benzylcoumarin derivatives already reported as GPR55 antagonists in the β-arrestin assay [12], some of which show neuroprotective properties in LPS-activated microglial cells [24]. The synthesized compounds were subjected to a radioligand displacement assay to determine their affinity towards GPR55 compared to CB1R and CB2R. The *N*-methylated compound **2** showed the greatest GPR55 affinity within the series, with a *K*_i_ of 1.2 nM. Compound **1**, the nor-analogue of **2**, showed complete selectivity over both CBRs (*K*_i_ > 10,000 nM) while preserving a high GPR55 affinity (*K*_i_ = 14 nM). Functional data, obtained by applying the p-ERK activation assay, showed a full GPR55 agonist profile for all the new 3-benzylquinolin-2(1*H*)-ones. The obtained results allowed us to outline some preliminary SAR for this type of molecule. In particular, the absence of a methyl substituent on the nitrogen of the central nucleus determines complete selectivity over CB2R without strongly reducing GPR55 affinity, while the presence of two methoxyl groups at positions 5 and 2′ ensures complete selectivity over the CB1R receptor. Compound **1**, in which the two advantageous structural features are simultaneously present, represents the most interesting 3-benzylquinolin-2(1*H*)-one derivative within this new series. Moreover, all four compounds were predicted to be able to cross the blood–brain barrier and to be well absorbed from the gastrointestinal tract.

The present work allowed the identification of new potent and selective agonists of the GPR55 receptor, which could be employed as useful tools for the in-depth study of this receptor. Moreover, this first small series of compounds will pave the way for the development of a new and broader class of derivatives based on the 3-benzylquinolin-2(1*H*)-one scaffold, which will allow one to extend the SAR for GPR55 ligands, in order to obtain more information about its binding site and to clarify its role as a therapeutic target, especially in the modulation of neuroinflammatory processes.

## Figures and Tables

**Table 1:** Radioligand binding assays on *h*GPR55 and *h*CBRs ^a^.

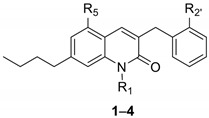						
Compound	R_1_	R_5_	R_2′_	*K*_i_ *h*GPR55 (nM)	*K*_i_ *h*CB_1_R (nM)	*K*_i_ *h*CB_2_R (nM)
CP55,940				13 (9.4–19)	12 (5.8–26)	128 (14–58)
O-1602				10 (7.6–14)	-	-
**1**	H	OCH_3_	OCH_3_	14 (8.1–24)	>10,000	>10,000
**2**	CH_3_	OCH_3_	OCH_3_	1.2 (0.58–3.3) *	>10,000	6.9 (2.4–17)
**3**	H	OH	OH	6.2 (4.6–8.4) *	5.1 (1.0–26)	>10,000
**4**	CH_3_	OH	OH	7.1 (6.0–8.4) *	29 (14–65)	7.9 (4.8–13)

^a^ Affinity (*K*_i_) was estimated for each compound using cell membranes (25 µg/sample) derived from CHO cells expressing either human GPR55, CB1R or CB2R. Membranes were incubated with 1 nM [^3^H]CP55,940 and 0.1–10 µM of each compound for 2 h and radioactivity was measured as previously described [27]. Data were fit to a 3-parameter non-linear regression in GraphPad (v. 9.0). Data are means with 95% confidence interval (CI). *n* = 3–4 independent experiments. Statistical analyses were by non-overlapping CI. * *p* < 0.05 relative to GPR55 within compound. Concentration-response curves are shown in Figure 3.

**Table 2:** p-ERK1/2 activation assay at *h*GPR55 ^a^.

Compound	EC_50_ 95% CI (nM)	*E*_max_ (Fold)
CP55,940	4.2 (1.2–12)	1.3 ± 0.06
O-1602	61 (19–190) *	1.3 ± 0.03
**1**	6.4 (0.38–48)	1.5 ± 0.06
**2**	1.1 (0.31–28)	1.4 ± 0.07
**3**	1.4 (0.15–22)	1.2 ± 0.04
**4**	11 (1.0–56)	1.3 ± 0.03

^a^ Potency (EC_50_) and efficacy (*E*_max_) were estimated for each compound in CHO cells expressing human GPR55 and treated with 0.1–10 µM compounds for 30 min. Data were fit to a 3-parameter non-linear regression in GraphPad (v. 9.0). Data are means with 95% CI (EC_50_) or means with SEM (*E*_max_). *n* = 6 independent experiments performed in duplicate. Statistical analyses were by non-overlapping CI. * *p* < 0.05 relative to CP55,940. Concentration-response curves are shown in Figure 4.

## Data Availability

Data is contained within the communication and Supplementary Material.

## Supplementary Materials

The following supporting information can be downloaded at: https://www.mdpi.com/article/10.3390/ph15070768/s1. Figure S1: ^1^H NMR and ^13^C NMR of compound **1**; Figure S2: ^1^H NMR and ^13^C NMR of compound **2**; Figure S3: ^1^H NMR and ^13^C NMR of compound **3**; Figure S4: ^1^H NMR and ^13^C NMR of compound **4**; Figure S5: NOESY spectrum of compound **14**; Figure S6: NOESY spectrum of compound **15**; Figure S7: HPLC data of compound **1**; Figure S8: HPLC data of compound **2**; Figure S9: HPLC data of compound **3**; Figure S10: HPLC data of compound **4**; Table S1: Average ligand root-mean-square deviation (RMSD) calculated during 100 ns of MD simulation for the eight different **2**-*h*GPR55 complexes analyzed; Table S2: Binding free energy values calculated for the eight different **2**-*h*GPR55 complexes using the MM-PBSA method.

## Abbreviations

The GPCR, G protein-coupled receptor; LPI, L-α-lysophosphatidylinositol; PLC, phospholipase C; ROCK, rho-associated protein kinase; RhoA, Ras homolog family member A; IP_3_, inositol 1,4,5-trisphosphate; ERK, extracellular signal-regulated kinase; MAP, mitogen-activated protein kinase; CBRs, cannabinoid receptors; CB1R, type 1 cannabinoid receptor; CB2R, type 2 cannabinoid receptor; Δ^9^-THC, Δ^9^-tetrahydrocannabinol; abn-CBD, abnormal cannabidiol; CHO, Chinese hamster ovary; PD, Parkinson’s disease; LPS, lipopolysaccharide; Pd_2_(dba)_3_, tris(dibenzylideneacetone)dipalladium(0); Ruphos, 2-dicyclohexylphosphino-2′,6′-diisopropoxybiphenyl; *t*-BuONa, sodium *tert*-butoxide; EtOH, ethanol; DMF, *N*,*N*-dimethylformamide; DCM, dichloromethane; MeOH, methanol; Et_3_N, triethylamine; AcOEt, ethyl acetate; DMSO-*d_6_*, deuterated dimethyl sulfoxide; NMR, nuclear magnetic resonance; ppm, part per million; s, singlet; d, doublet; dd, double doublet; t, triplet; dt, double triplet; bs, broad signal; m. p., melting point; HRMS, high resolution mass spectrometry; rt, room temperature; TLC, thin layer chromatography; UV, ultra violet; HPLC, high performance liquid chromatography; ACN, acetonitrile; MD, molecular dynamics; POPC, 1-palmitoyl-2-oleoyl-sn-glycero-3-phosphocholine; RMSD, root-mean-square deviation; MM-PBSA, Molecular Mechanics—Poisson-Boltzmann Surface Area; CG, conjugate gradient; GAFF, General Amber Force Field.
