# Supplementary material for: Novel Potent and Selective Agonists of the GPR55 Receptor Based on the 3-Benzylquinolin-2(1H)-One Scaffold
Source: Pharmaceuticals (Basel). 2022 Jun 21;15(7):768. doi: 10.3390/ph15070768 (PMC9320067; doi:10.3390/ph15070768)
Supplement: Supplementary file 1 [file pharmaceuticals-15-00768-s001.zip › pharmaceuticals-1747293-supplementary.pdf]

Supplementary Material

Novel potent and selective agonists of the GPR55 receptor based on the 3-benzylquinolin-2(1*H*)-one scaffold

Costanza Ceni [1,2], Michael J. Benko [3], Kawthar A. Mohamed [3], Giulio Poli [1], Miriana Di Stefano[1,2], Tiziano Tuccinardi [1], Maria Digiacomo [1], Massimo Valoti [4], Robert B. Laprairie [3,*], Marco Macchia [1] and Simone Bertini [1,*]

[1] Department of Pharmacy, University of Pisa, Via Bonanno Pisano 6, 56126, Pisa, Italy; costanza.ceni@phd.unipi.it (C.C.); giulio.poli@unipi.it (G.P.); miriana.distefano@phd.unipi.it (M.D.S.); tiziano.tuccinardi@unipi.it (T.T.); maria.digiacomo@unipi.it (M.D.); marco.macchia@unipi.it (M.M.)
[2] Doctoral School in Life Sciences, University of Siena, Via Aldo Moro 2, 53100, Siena, Italy
[3] College of Pharmacy and Nutrition, University of Saskatchewan, Saskatoon SK Canada; michael.benko@usask.ca (M.J.B.); kam913@mail.usask.ca (K.A.M.)
[4] Department of Life Sciences, University of Siena, Via Aldo Moro 2, 53100, Siena, Italy; massimo.valoti@unisi.it
* Correspondence: simone.bertini@unipi.it (S.B.); robert.laprairie@usask.ca (R.B.L)

Table of Contents

Figures S1-S4. ^{1}H NMR and ^{13}C NMR of compounds **1-4** …………………………….. S2-S5

Figures S5-S6. NOESY spectra of compounds **14** and **15** ……………………………… S6-S7

Figure S7-S10. HPLC Traces of compounds **1-4** ……………………………………. S8-S11

Table S1. RMSD analysis of the eight **2**-hGPR55 complexes during MD simulations … S12

Table S2. Binding free energies calculated for of the eight **2**-hGPR55 complexes …….. S13

Figure S1. ^{1}H NMR and ^{13}C NMR of compound **1**

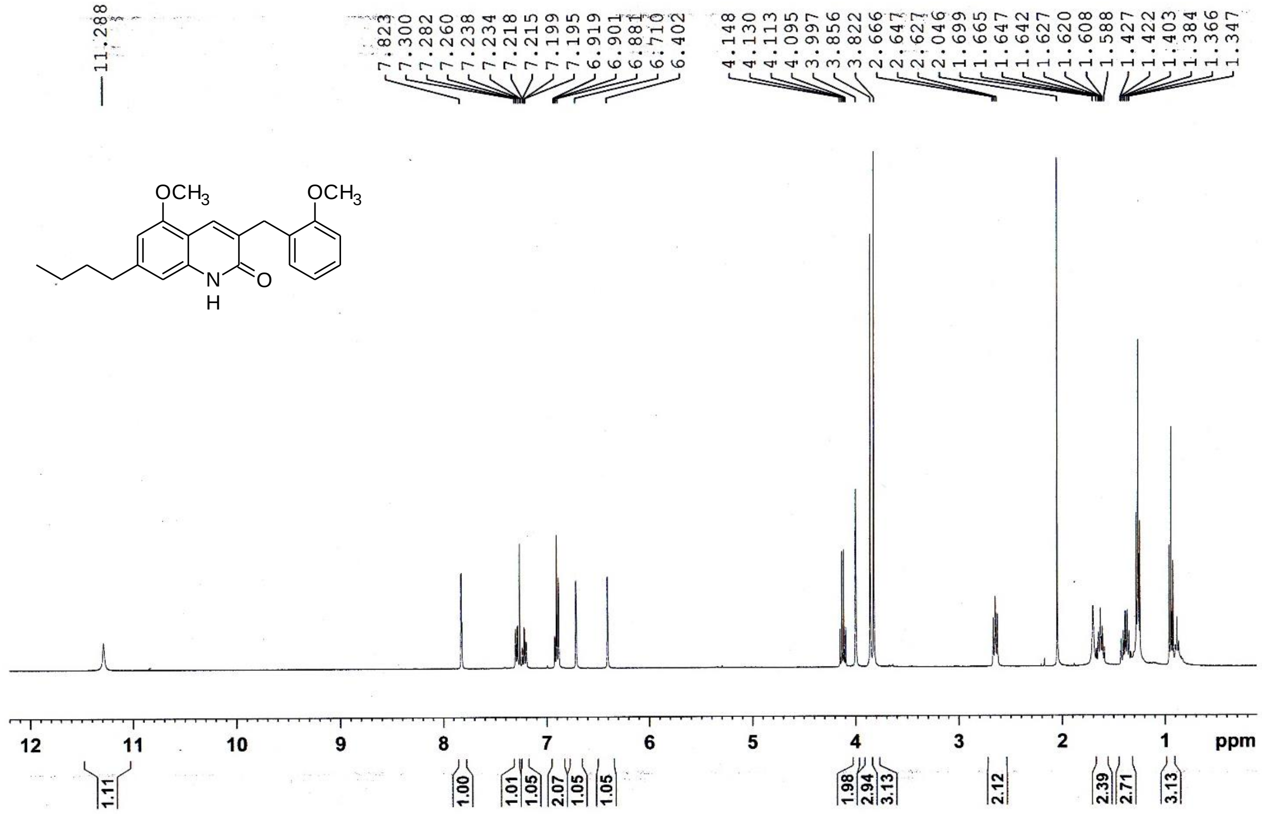

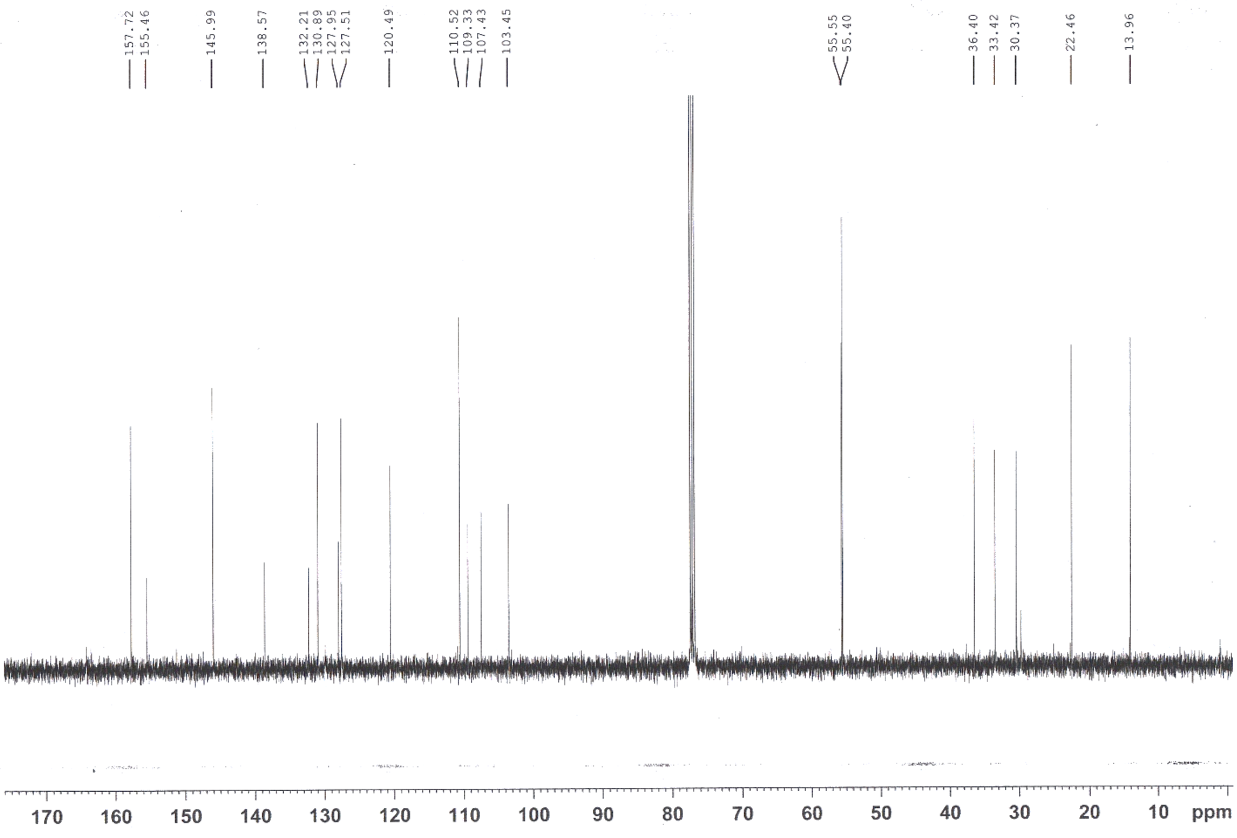

Figure S2. ^{1}H NMR and ^{13}C NMR of compound **2**

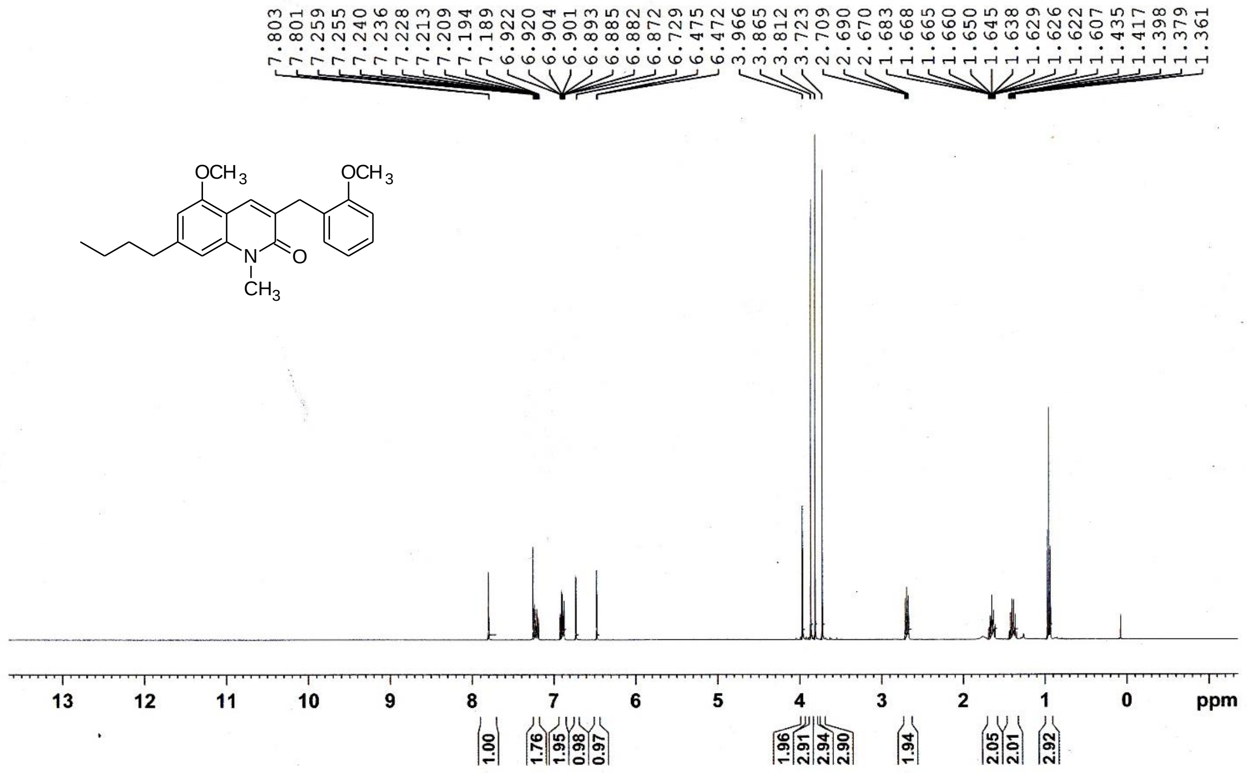

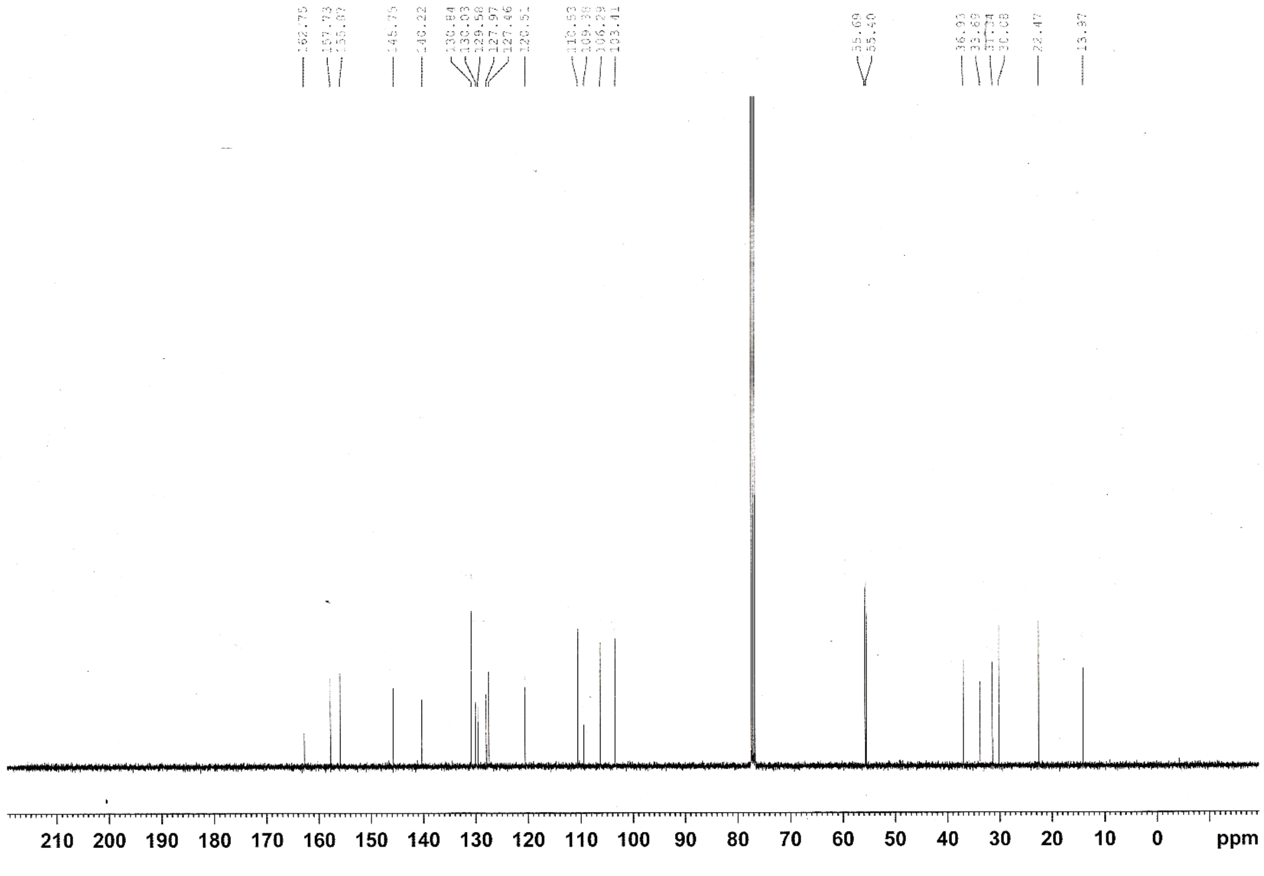

Figure S3. ^{1}H NMR and ^{13}C of compound **3**

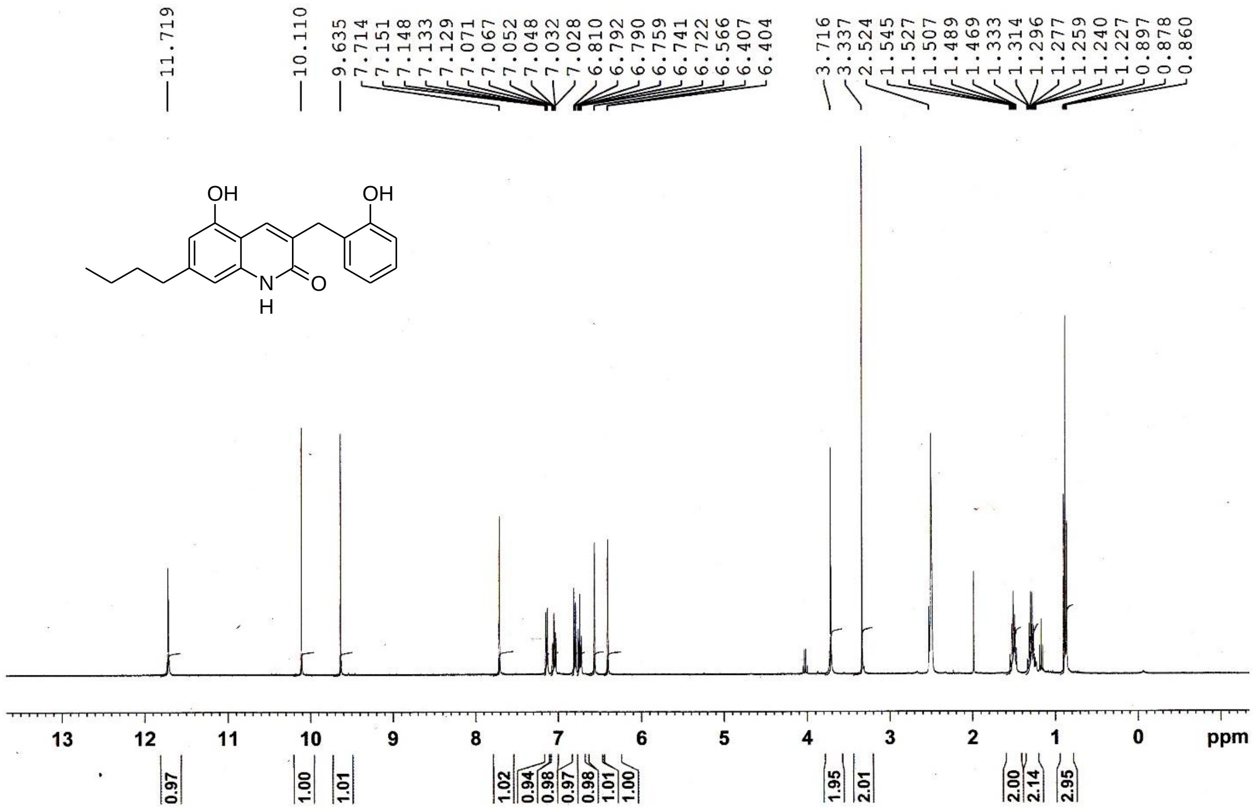

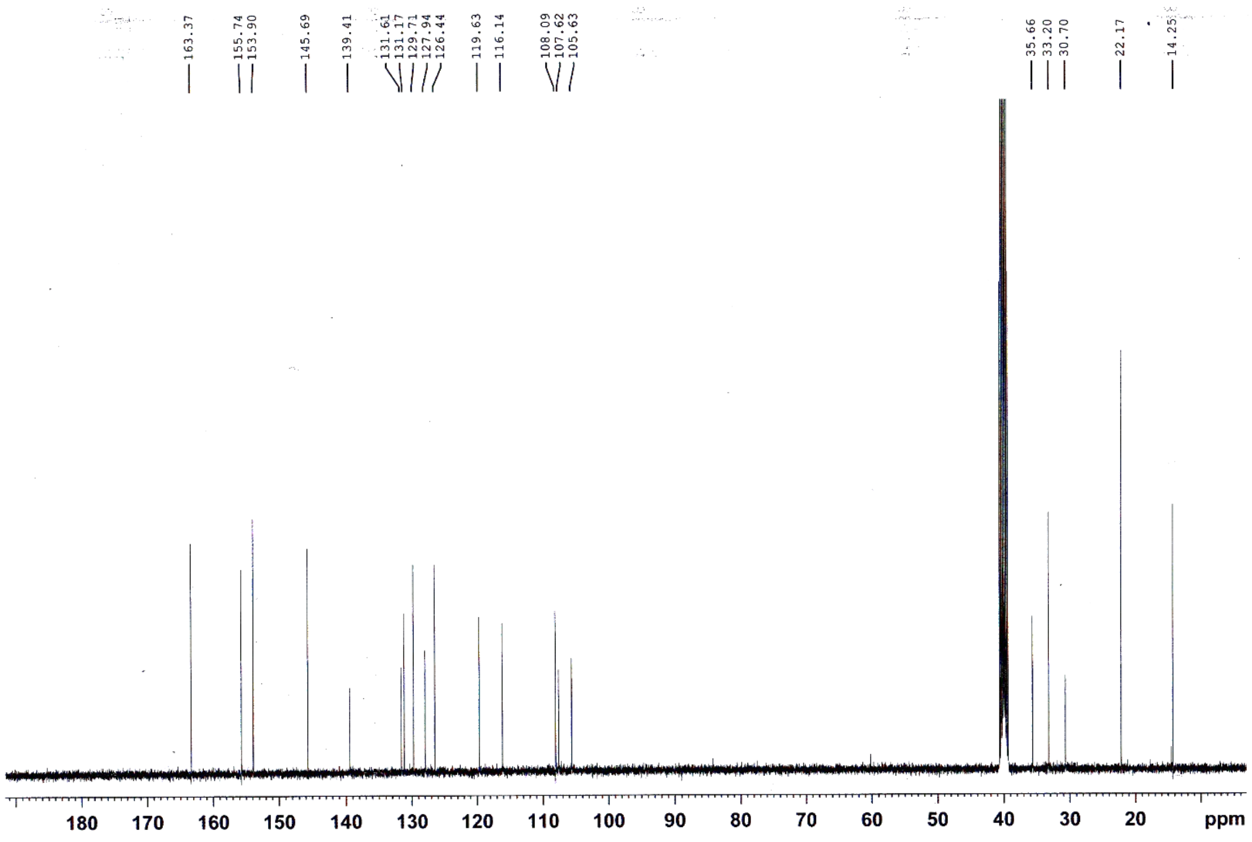

Figure S4. ^{1}H NMR and ^{13}C of compound **4**

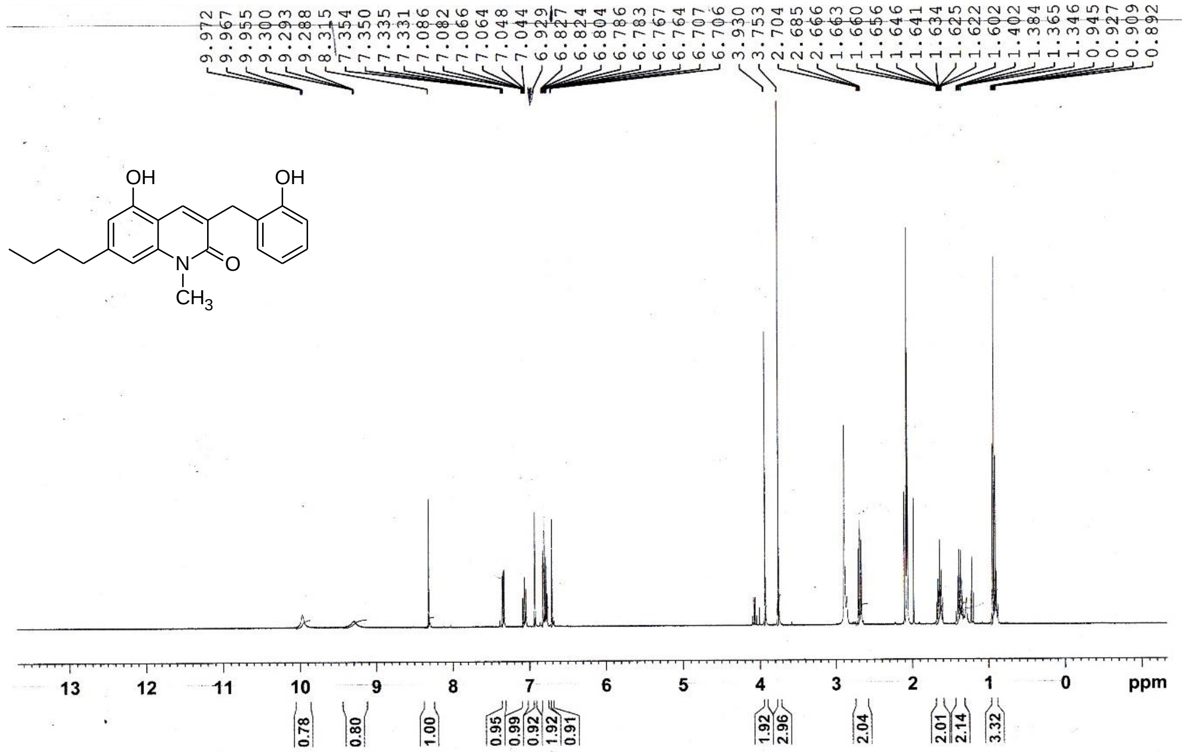

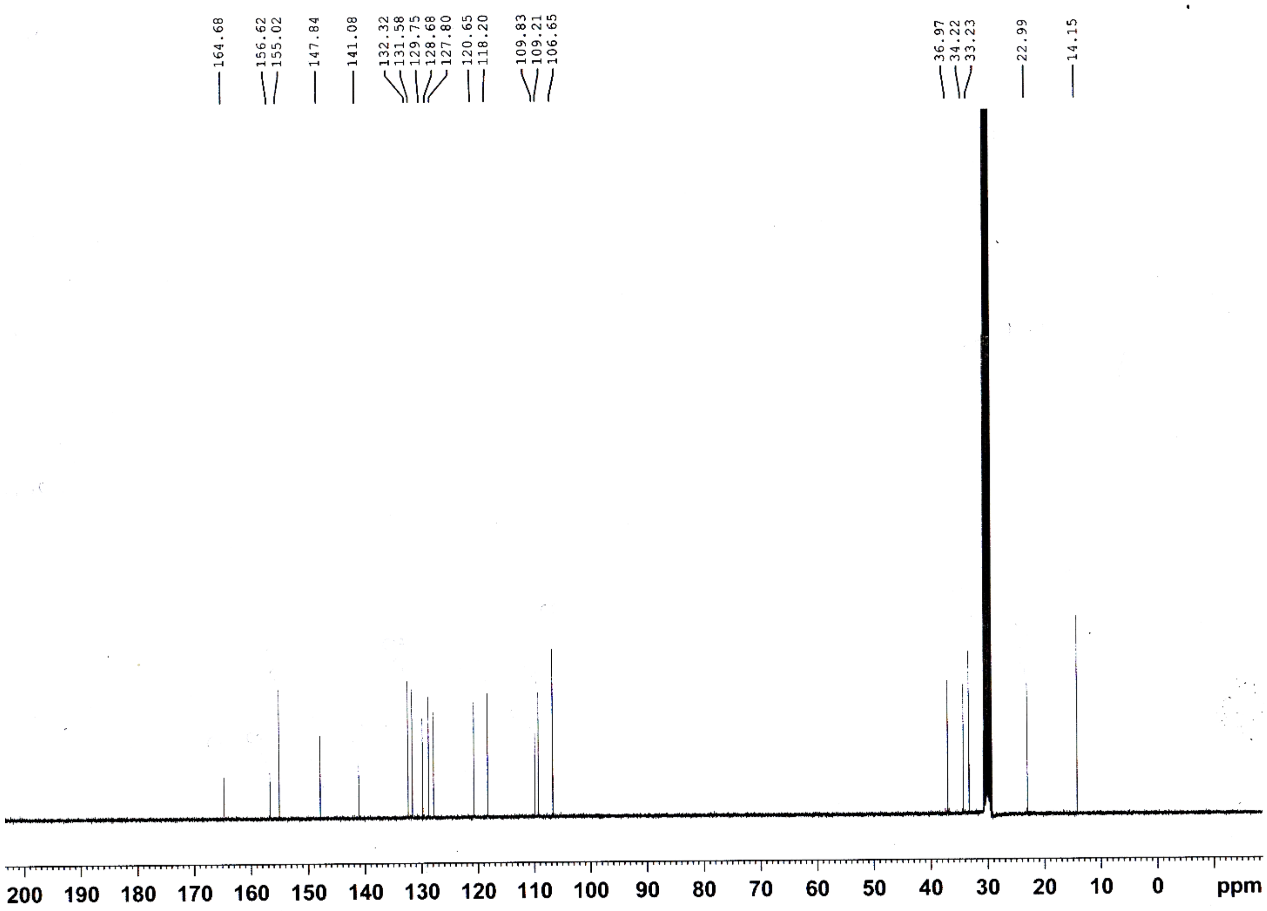

Figure S5. NOESY spectrum of compound **14**. Spatial interaction H_4/benzylic methylene (red circle) and H4/methoxy (5-position) (green circle) is observed. Only a slight spatial interaction H_4/CH_3 (4′′ position) (light blue circle) is present. The methylene group (1′′-position) interacts with both aromatic hydrogens H_6 and H_8 (purple circles). The methoxy group (5-position) interacts with H_6 (cyan circle), but not with H_8.

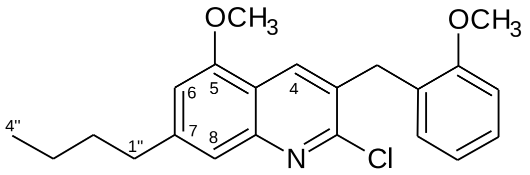

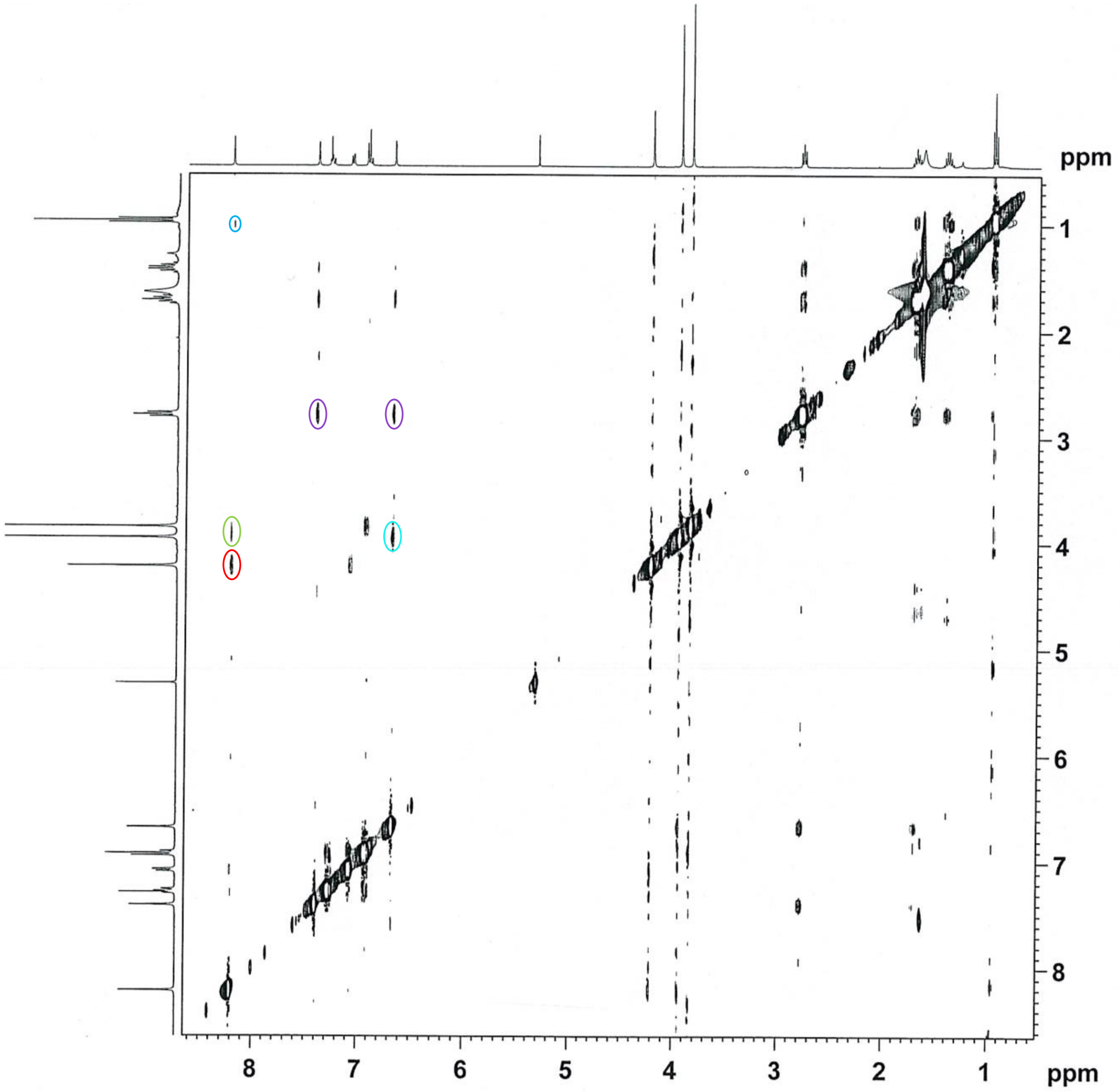

Figure S6. NOESY spectrum of compound **15**. Spatial interaction H_4/methylene (1′′-position) (green circle) and H_4/benzylic methylene (red circle) is observed. The H_6 proton interacts with the methylene (1′′- position) (light blue circle). The methoxy group (7-position) interacts with both H_6 and H_8 protons (purple circles).

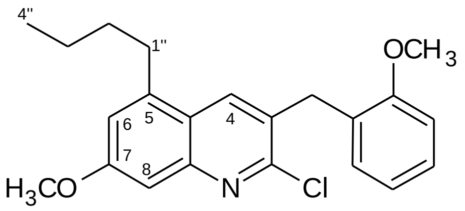

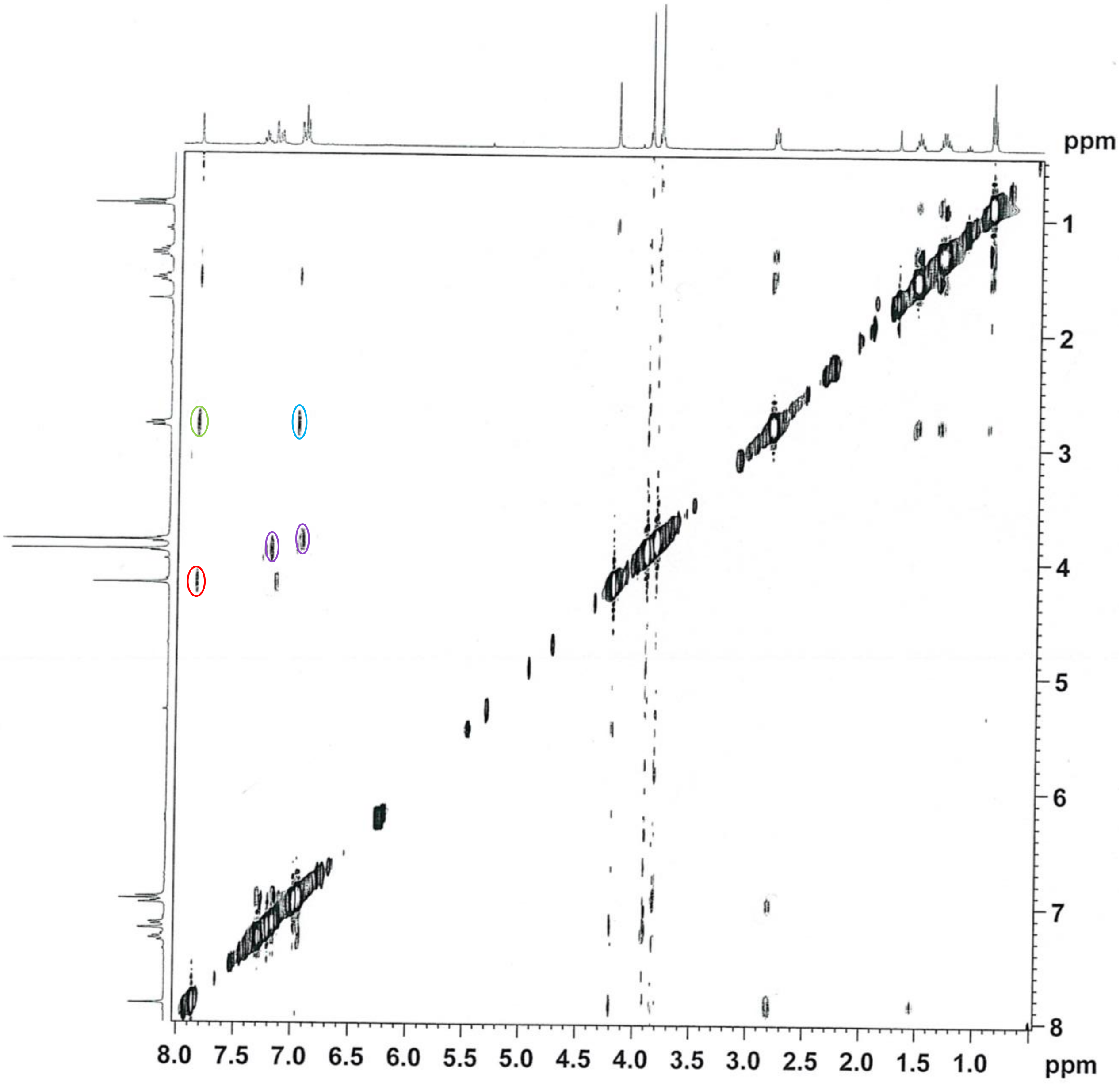

Figure S7. HPLC data of compound **1**.

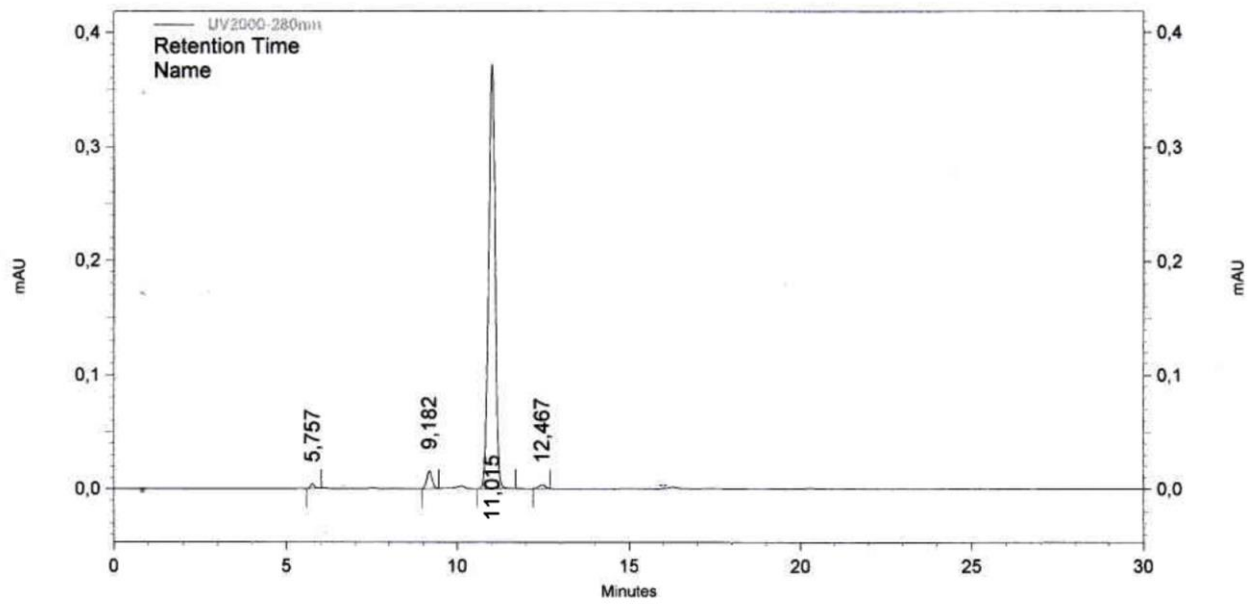

UV2000-280nm Results (System (07/09/2021 8.38.21) (Reprocessed))

Name	Retention Time	Area	Area Percent	Integration Codes
	5,757	30042	0,585	MM
	9,182	169788	3,306	MM
	11,015	4890624	95,214	MM
	12,467	45997	0,896	MM
Totals		5136451	100,000	

Figure S8. HPLC data of compound **2**.

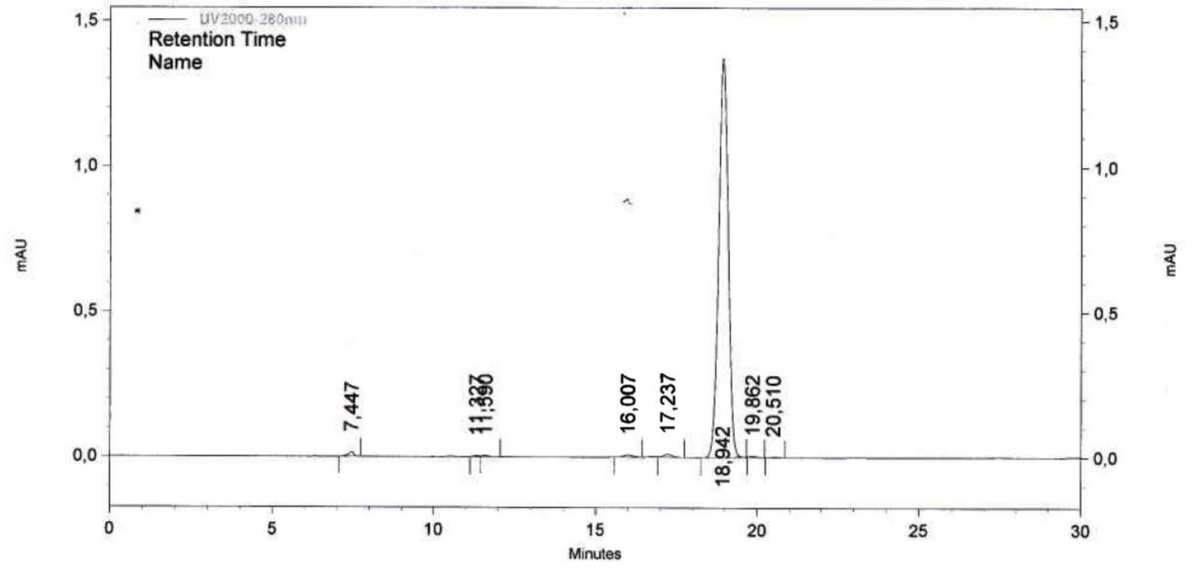

UV2000-280nm Results (System (18/11/2021 5.09.01) (Reprocessed))

Name	Retention Time	Area	Area Percent	Integration Codes
	7,447	177980	0,617	MM
	11,327	39119	0,136	MM
	11,590	61694	0,214	VV
	16,007	135213	0,469	MM
	17,237	165765	0,575	MM
	18,942	28174102	97,705	MM
	19,862	38407	0,133	MM
	20,510	43465	0,151	MM
Totals		28835745	100,000	

Figure S9. HPLC data of compound **3**.

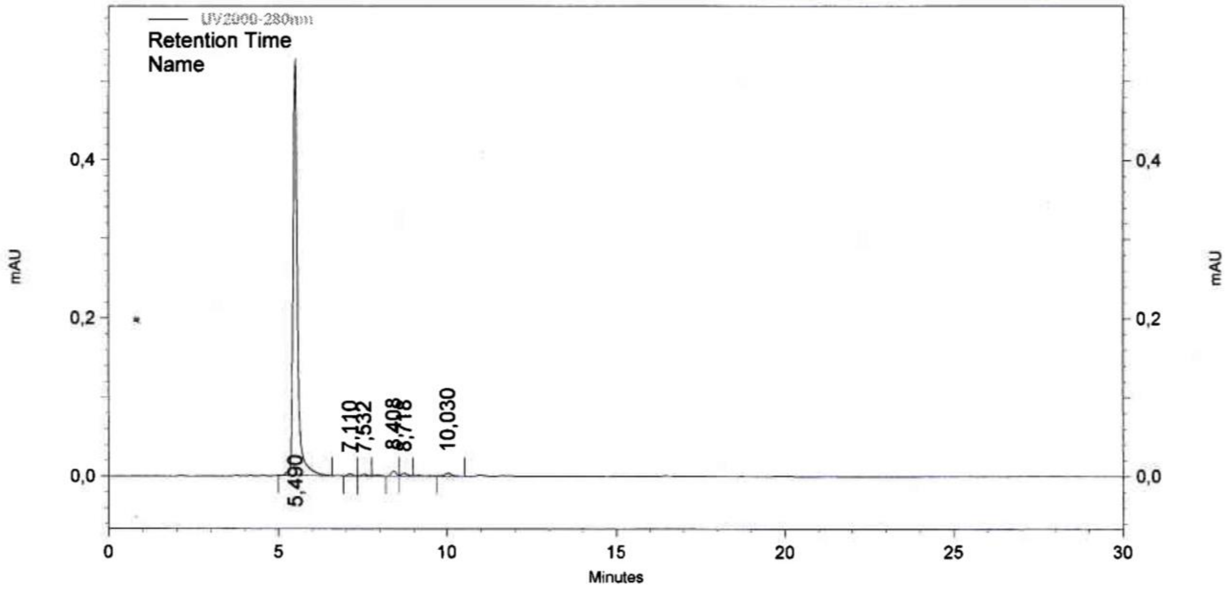

UV2000-280nm Results (System (07/09/2021 9.30.42) (Reprocessed))

Name	Retention Time	Area	Area Percent	Integration Codes
	5,490	4756943	95,194	MM
	7,110	34093	0,682	VV
	7,532	31695	0,634	VV
	8,408	68532	1,371	VV
	8,718	45633	0,913	VV
	10,030	60197	1,205	VV
Totals		4997093	100,000	

Figure S10. HPLC data of compound **4**.

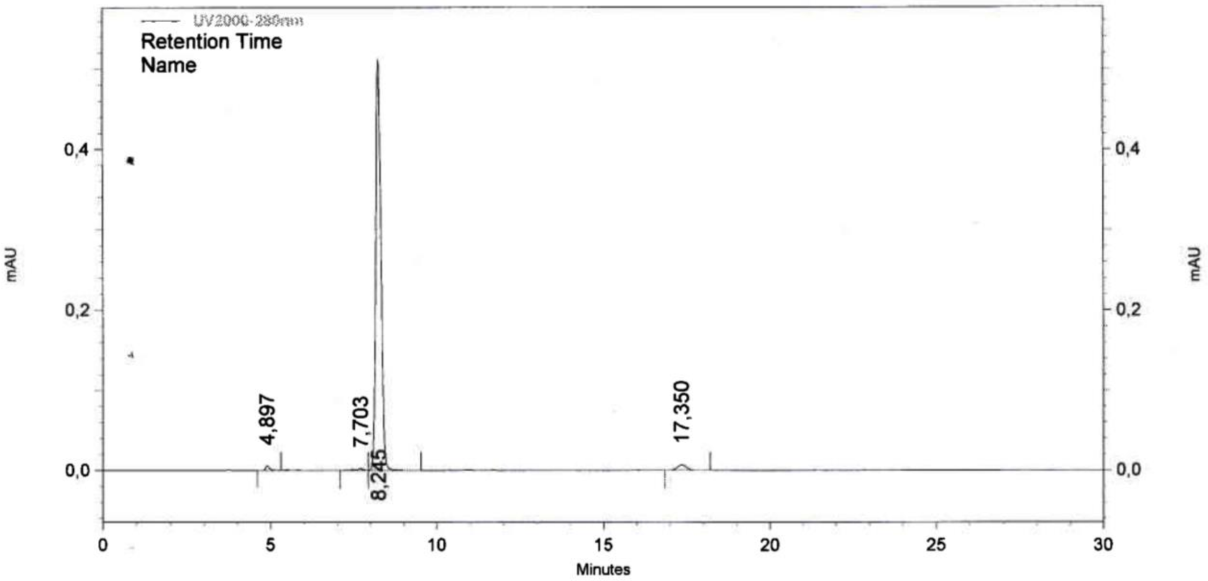

UV2000-280nm
Results (System
(07/09/2021
10.10.44)
(Reprocessed))

Name	Retention Time	Area	Area Percent	Integration Codes
	4,897	52323	0,917	VV
	7,703	38417	0,673	VV
	8,245	5485113	96,088	VV
	17,350	132600	2,323	BV
Totals		5708453	100,000	

Table S1. Average ligand root-mean-square deviation (RMSD) calculated during 100 ns of MD simulation for the eight different **2**-hGPR55 complexes analyzed.

Complex	Ligand RMSD
**C1**	4.0
**C2**	1.3
**C3**	0.9
**C4**	2.1
**C5**	4.9
**C6**	5.3
**C7**	2.2
**C8**	1.2

Table S2. Binding free energy values calculated for the eight different **2**-hGPR55 complexes using the MM-PBSA method (values are expressed in kcal/mol).

Complex	MM-PBSA binding energy (kcal/mol)
**C1**	-38.5
**C2**	-48.3
**C3**	-41.4
**C4**	-39.6
**C5**	-37.9
**C6**	-36.0
**C7**	-42.4
**C8**	-41.4
